# Correction: Genomic and bioacoustic variation in a midwife toad hybrid zone: A role for reinforcement?

**DOI:** 10.1371/journal.pone.0337571

**Published:** 2025-12-01

**Authors:** Johanna Ambu, Christophe Dufresnes

After the publication of this article [1], readers and the authors themselves contacted *PLOS One* about an error that was made in this study. The authors performed multivariate analyses of mating calls to test for reinforcement, based on a dataset of four bioacoustic variables, namely the dominant frequency (DF), the rising time (RT), the note duration (ND), and a fourth variable dubbed pulse rate (PR).

However, midwife toads do not have pulsed calls, and the fourth variable PR actually consists of the number of oscillations (not pulses) per time unit, and is therefore a manual calculation of DF. In effect, the multivariate analysis technically included three (not four) variables, with one variable (DF) being pseudo-replicated.

Re-analyses restricted to the three relevant variables yielded mostly similar patterns, as follows. The principal component analysis (PCA) emphasized the call differences between species, also retrieved using the multivariate analysis of variance (MANOVA; *F* = 5.2, *P* = 0.003). In the reanalysis, the authors also showed: (1) a significantly lower call overlap in the parapatric than in the allopatric populations; (2) no significant change in interspecific call differences between parapatric and allopatric populations; (3) significantly lower variation in parapatric than in the allopatric populations for *Alytes almogavarii* when considering the whole sampling, but not when down-sampling this species, as well as in *A. obstetricans*, and when cumulating both species. The corrected Fig 3 shows the corrected PCA and the corrected Table 1 provides the corrected test statistics. The only noteworthy change from the original results is that the *P*-values associated with the parapatric reduction of bioacoustic variation fall above the 0.05 threshold when down-sampling *A. almogavarii* and when both species are combined; these were only marginally significant in the original results (*P* = 0.03–0.05).

The conclusions regarding reinforcement in this hybrid zone thus remains unchanged, namely that the phenomenon may occur (according to the reduced interspecific call overlap in the hybrid zone), is seemingly not preferentially driven by character displacement (according to the similar levels of call differentiation in and outside the hybrid zone), but partly by a reduction in call diversity (according to the overall lower variation in one of the species). The origin of these bioacoustic patterns remain an open question, as discussed in the article and emphasized by its title [1]. The other main results, namely the location and patterns of introgression in the hybrid zone, are not affected.

The original article overlooked a previous bioacoustic study on a different pair of *Alytes* species, *Alytes obstetricans* and *Alytes cisternasii*, where patterns of sex-specific call preferences were shown to be consistent with reinforcement [81]. This study thus aligns with these previous findings in suggesting reinforcement as a relevant evolutionary process in this amphibian group.

An updated version of the dataset and scripts associated with this publication has been provided (doi: 10.5281/zenodo.15919562).

The Data Availability Statement is corrected as follows to incorporate the revised data repository DOI:

**Data Availability:** 16S sequences are available on GenBank (accessions OR725791–OR725973 and OR916149–OR916186); raw ddRAD sequence reads are archived on NCBI SRA under BioProject PRJNA949685; analyzed datasets (16S alignment, SNP data, bioacoustic data) are available on Zenodo (doi: 10.5281/zenodo.15919562).

Please see the corrected Table 1 and Fig 3 here:

The authors have provided the following corrections to the original text following the reanalysis of their dataset using the three bioacoustic variables, excluding the pseudoreplicated variable PR:

In the Materials and Methods section, the second paragraph under the subheading “Bioacoustic analyses” is corrected to:

“All recordings were processed in Raven Pro 1.6.1 (The Cornell Lab, Ithaca, NY, USA). Three parameters were measured on each note and averaged by individual: (1) the dominant frequency DF (Hz), as the frequency of the greatest energy in the note; (2) the note duration ND (s), as the time between the first and last pulses of the note, (3) the rising time RT (s), as the time between the first pulse and the pulse with the highest frequency. See S1 Fig for a graphical display.”

In the Materials and Methods section, the fourth sentence in the fourth paragraph under the subheading “Bioacoustic analyses” is corrected to: “This was done separately for three pairs of PCs (PC1/PC2, PC1/PC3, PC2/PC3), and averaged over PCs, weighing for their relative contributions.”

In the Results and Discussion section, the third paragraph is corrected to:

“In parallel, we found significant differences in the mating calls of the two species (MANOVA, *F* = 5.2, dof = 1, *P* = 0.003). These differences are shown by the first components of the PCA (Fig 3), namely PC1, which reflects the note duration (ND) and the rising time (RT) – both being generally higher in *A. obstetricans* – and PC2, which reflects the dominant frequency (DF) – generally higher in *A. almogavarii* (S4 Table). The two species’ calls significantly overlap less in the parapatric than the allopatric populations (Table 1, Fig 3). However, this reduced overlap is not particularly due to a shift in the average call parameters: the interspecific call differences are not significantly greater between the parapatric and allopatric populations (Table 1). In parallel, the parapatric individuals of *A. almogavarii* features significantly lesser call variation than the allopatric individuals, a trend that is however absent for *A. obstetricans*, and consequently not significant when combining both species together (Table 1). Generally similar patterns are obtained when the most represented groups are down-sampled in the permutation analyses, with the reduction of standard deviation in *A. almogavarii* falling slightly above the 0.05 significant threshold (Table 1).”

In the Results and Discussion section, the second sentence of the fourth paragraph is corrected to: “In addition, the dissimilarities reported here may be associated with a reduction of standing variation in one of the species (Fig 1, right) rather than a disruptive character displacement (Fig 1, left).”

The authors also provided the following correction to incorporate a discussion of a related study [81]. In the Results and Discussion section, the sixth and final paragraph is corrected to:

“To date, reinforcement has been primarily examined in reproductively well-isolated species that form bimodal hybrid zones, i.e., where hybridization and introgression is drastically restricted, and distinct hybrid/parental categories co-exist. Some of these cases have been criticized because character displacement between deeply-diverged species can be explained by alternative processes (e.g., ecological adaptations that evolved before the contact [5, 79]; runaway sexual selection [80]). In midwife toads, shifts in sex-specific call preferences have been interpreted as evidence of reinforcement between such distantly related species, namely *Alytes obstetricans* and *A. cisternasii* [81]. Our study aligns with these previous findings in suggesting reinforcement as a relevant evolutionary process in *Alytes*. It further highlights that younger, lesser differentiated species that form unimodal hybrid zones, i.e., where large parts of the genomes remain permeable to gene flow, can also in principle feature the genetic and phenotypic variation necessary to evolve reinforcement, even without obvious character displacement. The empirical focus on such systems should thus widen our understanding of the phenomenon across broader evolutionary contexts.”

Updated, correct versions of S1 Fig and S4 Table are provided with this Correction notice.

**Table 1:** Comparison of the bioacoustic variation between allopatric and parapatric species across the four axes of the PCA (Fig 3).

	allopatric	parapatric	∆	*P*
Overlap between species	1.28	0.55	–0.73	**0.032** (**0.005**)
Centroid distance between species	0.98	1.54	+0.55	0.201 (0.117)
Standard deviation – *obstetricans*	2.76	2.81	+0.05	0.608 (0.546)
Standard deviation – *almogavarii*	3.12	2.25	–0.87	**0.032** (0.074)
Standard deviation – cumulated	5.88	5.06	–0.82	0.160 (0.192)

*P* indicates the proportion of the null distribution of ∆ that exceeds the observed ∆ (in absolute values); in bold when significant; brackets: *P* when down-sampling all groups to 10 individuals. For instance, only 3.2% (*P* = 0.032) of the 1,000 permutations yielded a greater allopatric-parapatric difference than the observed difference of 0.73, suggesting statistical significance.

**Fig 3:**
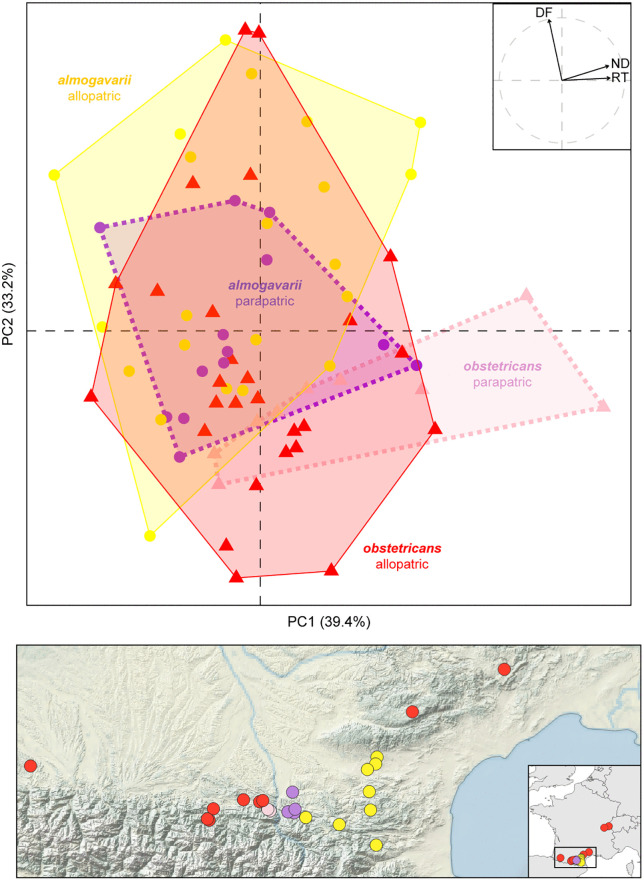
Bioacoustic variation among 71 individuals in (parapatric) and out (allopatric) of the hybrid zone. **(A)** PCA on three mating call parameters. **(B)** Geographic origin and group assignments of calls.

## Supporting information

S1 Fig Oscillogram (top) and spectrogram (bottom) of a note, showing the variables measured. (DOCX)

S4 Table Mean, standard variation (±) and range (in brackets) of the bioacoustic variables in the four groups. DF: dominant frequency; ND: note duration; RT: rising time.
(DOCX)
