# Supplementary material for: Genomic and bioacoustic variation in a midwife toad hybrid zone: A role for reinforcement?
Source: PLoS One. 2024 Nov 25;19(11):e0314477. doi: 10.1371/journal.pone.0314477 (PMC11588267; doi:10.1371/journal.pone.0314477)
Supplement: S1 Table — Column # indicates locality number as in Fig 1 (bold: transect samples; * reference samples to determine species-diagnostic RAD loci). Columns Y and X indicate latitude and longitude. Columns 16S and ddRAD-seq indicate individual accession numbers on GenBank and NCBI Sequence Read Archive (SRA). Column Call indicates individuals recorded for bioacoustic analyses, with reference to the online repository. (DOCX) [file pone.0314477.s001.docx]

**S1 Table. Details on the samples included in this study.** Column # indicates locality number as in Fig. 1 (bold: transect samples; * reference samples to determine species-diagnostic RAD loci). Columns Y and X indicate latitude and longitude. Columns 16S and ddRAD-seq indicate individual accession numbers on GenBank and NCBI Sequence Read Archive (SRA). Column Call indicates individuals recorded for bioacoustic analyses, with reference to the online repository.

| **#** | **Sample / Voucher** | **Species** | **Mitogroup** | **Locality** | **Y** | **X** | **16S** | **ddRAD-seq** | **Call** |
| --- | --- | --- | --- | --- | --- | --- | --- | --- | --- |
| **1*** | AVE01 | obstetricans | obstetricans | Avezac, France | 43.06645 | 0.337807 | OR725801 | SRR31341061 |  |
| **1** | AVE02 | obstetricans | obstetricans | Avezac, France | 43.06645 | 0.337807 | OR725802 |  |  |
| **1** | AVE05 | obstetricans | obstetricans | Avezac, France | 43.06645 | 0.337807 | OR725803 |  |  |
| **1*** | AVE06 | obstetricans |  | Avezac, France | 43.06645 | 0.337807 |  | SRR31341050 |  |
| **1*** | AVE07 | obstetricans |  | Avezac, France | 43.06645 | 0.337807 |  | SRR31341039 |  |
| **2*** | GAL01 | obstetricans |  | Mont-de-Galié, France | 42.98972 | 0.647247 |  | SRR31341045 |  |
| **2** | GAL03 | obstetricans | obstetricans | Mont-de-Galié, France | 42.98972 | 0.647247 | OR725871 |  |  |
| **2*** | GAL04 | obstetricans |  | Mont-de-Galié, France | 42.98972 | 0.647247 |  | SRR31341044 |  |
| **2*** | GAL05 | obstetricans |  | Mont-de-Galié, France | 42.98972 | 0.647247 |  | SRR31341043 |  |
| **3*** | CHAC01 | obstetricans | obstetricans | Saleich, France | 43.03515 | 0.965823 | OR725832 | SRR31341068 |  |
| **3*** | CHAC02 | obstetricans | obstetricans | Saleich, France | 43.03515 | 0.965823 | OR725833 | SRR31341067 |  |
| **3** | CHAC03 | obstetricans | obstetricans | Saleich, France | 43.03515 | 0.965823 | OR725834 |  |  |
| **3*** | CHAC04 | obstetricans | obstetricans | Saleich, France | 43.03515 | 0.965823 | OR725835 | SRR31341066 |  |
| **3*** | CHAC05 | obstetricans | obstetricans | Saleich, France | 43.03515 | 0.965823 | OR725836 | SRR31341065 |  |
| **3** | CHAC06 | obstetricans | obstetricans | Saleich, France | 43.03515 | 0.965823 | OR725837 |  |  |
| **4** | BET01 | obstetricans | obstetricans | Samortein, France | 42.88917 | 1.05645 | OR725811 | SRR31341081 |  |
| **4** | BET02 | obstetricans | obstetricans | Samortein, France | 42.88917 | 1.05645 | OR725812 | SRR31341080 |  |
| **4** | BET03 | obstetricans | obstetricans | Samortein, France | 42.88917 | 1.05645 | OR725813 |  |  |
| **4** | BET05 | obstetricans |  | Samortein, France | 42.88917 | 1.05645 |  |  | 1 |
| **4** | BET06 | obstetricans |  | Samortein, France | 42.88917 | 1.05645 |  |  | 1 |
| **4** | BET07 | obstetricans |  | Samortein, France | 42.88917 | 1.05645 |  |  | 1 |
| 5 | MNCN81020 | obstetricans | obstetricans | Etang de Lers, France | 42.80731 | 1.382611 | OR725897 | SRR31341029 |  |
| 5 | MNCN81021 | obstetricans | obstetricans | Etang de Lers, France | 42.80731 | 1.382611 | OR725898 | SRR31341027 |  |
| **6** | SDS01 | obstetricans | obstetricans | Sentenac-de-Sérou, France | 42.97258 | 1.388478 | OR725939 | SRR31341428 |  |
| **6** | SDS02 | obstetricans | obstetricans | Sentenac-de-Sérou, France | 42.97258 | 1.388478 | OR725940 | SRR31341427 |  |
| **6** | SDS03 | obstetricans | obstetricans | Sentenac-de-Sérou, France | 42.97258 | 1.388478 | OR725941 | SRR31341426 |  |
| **6** | SDS04 | obstetricans | obstetricans | Sentenac-de-Sérou, France | 42.97258 | 1.388478 | OR725942 |  |  |
| **6** | SDS05 | obstetricans | obstetricans | Sentenac-de-Sérou, France | 42.97258 | 1.388478 | OR725943 |  |  |
| **7** | VIE01 | obstetricans (admixed) | obstetricans | Montels, France | 43.00461 | 1.472231 | OR725964 | SRR31341434 |  |
| **7** | VIE02 | obstetricans (admixed) | obstetricans | Montels, France | 43.00461 | 1.472231 | OR725965 | SRR31341433 |  |
| **7** | VIE03 | obstetricans (admixed) | obstetricans | Montels, France | 43.00461 | 1.472231 | OR725966 | SRR31341432 |  |
| **7** | VIE04 | obstetricans (admixed) | obstetricans | Montels, France | 43.00461 | 1.472231 | OR725967 | SRR31341431 |  |
| **7** | VIE05 | obstetricans (admixed) | obstetricans | Montels, France | 43.00461 | 1.472231 | OR725968 | SRR31341430 |  |
| **7** | VIE06 | obstetricans (admixed) | obstetricans | Montels, France | 43.00461 | 1.472231 | OR725969 | SRR31341429 |  |
| **7** | VIE07 | obstetricans (admixed) | obstetricans | Montels, France | 43.00461 | 1.472231 | OR725970 |  |  |
| **7** | VIE08 | obstetricans (admixed) | obstetricans | Montels, France | 43.00461 | 1.472231 | OR725971 |  |  |
| **7** | VIE09 | obstetricans (admixed) | obstetricans | Montels, France | 43.00461 | 1.472231 | OR725972 |  |  |
| **7** | VIE22_01 | obstetricans (admixed) | obstetricans | Montels, France | 43.00461 | 1.472231 | OR725973 |  | 1 |
| **7** | VIE22_02 | obstetricans (admixed) |  | Montels, France | 43.00461 | 1.472231 |  |  | 1 |
| **7** | VIE23_01 | obstetricans (admixed) |  | Montels, France | 43.00461 | 1.472231 |  |  | 1 |
| **7** | VIE23_02 | obstetricans (admixed) |  | Montels, France | 43.00461 | 1.472231 |  |  | 1 |
| **7** | VIE23_03 | obstetricans (admixed) |  | Montels, France | 43.00461 | 1.472231 |  |  | 1 |
| **7** | VIE23_04 | obstetricans (admixed) |  | Montels, France | 43.00461 | 1.472231 |  |  | 1 |
| **8** | RECO01 | obstetricans (admixed) | almogavarii | Recort, France | 42.95737 | 1.538699 | OR725913 | SRR31341022 |  |
| **8** | RECO03 | obstetricans (admixed) | almogavarii | Recort, France | 42.95737 | 1.538699 | OR725914 | SRR31341021 |  |
| **8** | RECO05 | obstetricans (admixed) | almogavarii | Recort, France | 42.95737 | 1.538699 | OR725915 |  |  |
| **8** | RECO06 | obstetricans (admixed) | obstetricans | Recort, France | 42.95737 | 1.538699 | OR725916 |  | 1 |
| **8** | RECO07 | obstetricans (admixed) |  | Recort, France | 42.95737 | 1.538699 |  |  | 1 |
| **8** | RECO08 | obstetricans (admixed) |  | Recort, France | 42.95737 | 1.538699 |  |  | 1 |
| **8** | RECO09 | obstetricans (admixed) |  | Recort, France | 42.95737 | 1.538699 |  |  | 1 |
| **8** | RECO11 | obstetricans (admixed) |  | Recort, France | 42.95737 | 1.538699 |  |  | 1 |
| **8** | RECO12 | obstetricans (admixed) |  | Recort, France | 42.95737 | 1.538699 |  |  | 1 |
| **8** | RECO13 | obstetricans (admixed) |  | Recort, France | 42.95737 | 1.538699 |  |  | 1 |
| **8** | RECO14 | obstetricans (admixed) |  | Recort, France | 42.95737 | 1.538699 |  |  | 1 |
| **9** | BLAN01 | obstetricans (admixed) | obstetricans | Ganac, France | 42.94792 | 1.558236 | OR725821 | SRR31341076 |  |
| **9** | BLAN02 | obstetricans (admixed) | obstetricans | Ganac, France | 42.94792 | 1.558236 | OR725822 | SRR31341075 |  |
| **9** | BLAN03 | obstetricans (admixed) |  | Ganac, France | 42.94792 | 1.558236 |  | SRR31341074 |  |
| **9** | BLAN04 | obstetricans (admixed) | obstetricans | Ganac, France | 42.94792 | 1.558236 | OR725823 |  |  |
| **9** | BLAN05 | obstetricans (admixed) | obstetricans | Ganac, France | 42.94792 | 1.558236 | OR725824 |  |  |
| **9** | BLAN08 | obstetricans (admixed) |  | Ganac, France | 42.94792 | 1.558236 |  |  | 1 |
| **10** | BECQ01 | obstetricans (admixed) | almogavarii | Becq, France | 42.94497 | 1.57891 | OR725804 | SRR31341028 |  |
| **10** | BECQ02 | obstetricans (admixed) | obstetricans | Becq, France | 42.94497 | 1.57891 | OR725805 | SRR31341017 |  |
| **10** | BECQ03 | obstetricans (admixed) | obstetricans | Becq, France | 42.94497 | 1.57891 | OR725806 | SRR31341013 |  |
| **10** | BECQ04 | obstetricans (admixed) | obstetricans | Becq, France | 42.94497 | 1.57891 | OR725807 |  |  |
| **11** | CARA01 | almogavarii (admixed) | almogavarii | Caraybat, France | 42.94187 | 1.671247 | OR725826 | SRR31341071 |  |
| **11** | CARA02 | almogavarii (admixed) | almogavarii | Caraybat, France | 42.94187 | 1.671247 | OR725827 | SRR31341070 |  |
| **11** | CARA03 | almogavarii (admixed) | almogavarii | Caraybat, France | 42.94187 | 1.671247 | OR725828 | SRR31341069 |  |
| **11** | CARA04 | almogavarii (admixed) | almogavarii | Caraybat, France | 42.94187 | 1.671247 | OR725829 |  |  |
| **11** | CARA05 | almogavarii (admixed) | almogavarii | Caraybat, France | 42.94187 | 1.671247 | OR725830 |  |  |
| **12** | CIR01 | almogavarii (admixed) | almogavarii | Saint-Cirac, France | 42.94161 | 1.70461 | OR725838 | SRR31341064 |  |
| **12** | CIR02 | almogavarii (admixed) | almogavarii | Saint-Cirac, France | 42.94161 | 1.70461 | OR725839 | SRR31341063 |  |
| **12** | CIR03 | almogavarii (admixed) | almogavarii | Saint-Cirac, France | 42.94161 | 1.70461 | OR725840 |  |  |
| **12** | CIR04 | almogavarii (admixed) | almogavarii | Saint-Cirac, France | 42.94161 | 1.70461 | OR725841 | SRR31341062 |  |
| **12** | CIR05 | almogavarii (admixed) | almogavarii | Saint-Cirac, France | 42.94161 | 1.70461 | OR725842 | SRR31341060 |  |
| **12** | CIR06 | almogavarii (admixed) | almogavarii | Saint-Cirac, France | 42.94161 | 1.70461 | OR725843 | SRR31341059 |  |
| **12** | CIR07 | almogavarii (admixed) | almogavarii | Saint-Cirac, France | 42.94161 | 1.70461 | OR725844 |  |  |
| **12** | CIR08 | almogavarii (admixed) | almogavarii | Saint-Cirac, France | 42.94161 | 1.70461 | OR725845 |  |  |
| **12** | CIR09 | almogavarii (admixed) | almogavarii | Saint-Cirac, France | 42.94161 | 1.70461 | OR725846 |  |  |
| **12** | CIR22_01 | almogavarii (admixed) |  | Saint-Cirac, France | 42.94161 | 1.70461 |  |  | 1 |
| **12** | CIR22_02 | almogavarii (admixed) |  | Saint-Cirac, France | 42.94161 | 1.70461 |  |  | 1 |
| **12** | CIR23_01 | almogavarii (admixed) |  | Saint-Cirac, France | 42.94161 | 1.70461 |  |  | 1 |
| **12** | CIR23_02 | almogavarii (admixed) |  | Saint-Cirac, France | 42.94161 | 1.70461 |  |  | 1 |
| **12** | CIR23_03 | almogavarii (admixed) |  | Saint-Cirac, France | 42.94161 | 1.70461 |  |  | 1 |
| **12** | CIR23_04 | almogavarii (admixed) |  | Saint-Cirac, France | 42.94161 | 1.70461 |  |  | 1 |
| **13** | ROQ01 | almogavarii (admixed) | almogavarii | Roquefixade, France | 42.93553 | 1.755277 | OR725922 | SRR31341019 |  |
| **13** | ROQ02 | almogavarii (admixed) | almogavarii | Roquefixade, France | 42.93553 | 1.755277 | OR725923 | SRR31341018 |  |
| **13** | ROQ03 | almogavarii (admixed) | almogavarii | Roquefixade, France | 42.93553 | 1.755277 | OR725924 |  |  |
| **13** | ROQ05 | almogavarii (admixed) | almogavarii | Roquefixade, France | 42.93553 | 1.755277 | OR725925 | SRR31341016 |  |
| **13** | ROQ06 | almogavarii (admixed) | almogavarii | Roquefixade, France | 42.93553 | 1.755277 | OR725926 | SRR31341015 |  |
| **13** | ROQ07 | almogavarii (admixed) | almogavarii | Roquefixade, France | 42.93553 | 1.755277 | OR725927 | SRR31341014 |  |
| **13** | ROQ08 | almogavarii (admixed) | almogavarii | Roquefixade, France | 42.93553 | 1.755277 | OR725928 |  |  |
| **13** | ROQ09 | almogavarii (admixed) | almogavarii | Roquefixade, France | 42.93553 | 1.755277 | OR725929 |  |  |
| **13** | ROQ22_01 | almogavarii (admixed) |  | Roquefixade, France | 42.93553 | 1.755277 |  |  | 1 |
| **13** | ROQ22_02 | almogavarii (admixed) |  | Roquefixade, France | 42.93553 | 1.755277 |  |  | 1 |
| **13** | ROQ22_03 | almogavarii (admixed) |  | Roquefixade, France | 42.93553 | 1.755277 |  |  | 1 |
| **13** | ROQ22_04 | almogavarii (admixed) |  | Roquefixade, France | 42.93553 | 1.755277 |  |  | 1 |
| **13** | ROQ22_05 | almogavarii (admixed) |  | Roquefixade, France | 42.93553 | 1.755277 |  |  | 1 |
| **14** | BEN01 | almogavarii (admixed) | almogavarii | Benaix, France | 42.90614 | 1.85062 | OR725808 | SRR31341012 |  |
| **14** | BEN02 | almogavarii (admixed) | almogavarii | Benaix, France | 42.90614 | 1.85062 | OR725809 |  |  |
| **14** | BEN03 | almogavarii (admixed) | almogavarii | Benaix, France | 42.90614 | 1.85062 | OR725810 | SRR31341082 |  |
| **14** | BEN04 | almogavarii (admixed) |  | Benaix, France | 42.90614 | 1.85062 |  |  | 1 |
| **14** | BEN05 | almogavarii (admixed) |  | Benaix, France | 42.90614 | 1.85062 |  |  | 1 |
| **14** | BEN06 | almogavarii (admixed) |  | Benaix, France | 42.90614 | 1.85062 |  |  | 1 |
| 15 | PEC02 | almogavarii (admixed) | almogavarii | Perles, France | 42.74419 | 1.786684 | OR725900 | SRR31341026 |  |
| 15 | PEC03 | almogavarii (admixed) | almogavarii | Perles, France | 42.74419 | 1.786684 | OR725901 | SRR31341025 |  |
| 15 | PEC04 | almogavarii (admixed) | almogavarii | Perles, France | 42.74419 | 1.786684 | OR725902 |  |  |
| 15 | PEC05 | almogavarii (admixed) | almogavarii | Perles, France | 42.74419 | 1.786684 | OR725903 |  |  |
| 15 | PEC06 | almogavarii (admixed) | almogavarii | Perles, France | 42.74419 | 1.786684 | OR725904 |  |  |
| 15 | PEC07 | almogavarii (admixed) | almogavarii | Perles, France | 42.74419 | 1.786684 | OR725905 |  |  |
| 15 | PEC09 | almogavarii (admixed) | almogavarii | Perles, France | 42.74419 | 1.786684 | OR725906 |  |  |
| **16** | MAL01 | almogavarii | almogavarii | Mazuby, France | 42.80196 | 2.033066 | OR725888 | SRR31341033 |  |
| **16** | MAL02 | almogavarii | almogavarii | Mazuby, France | 42.80196 | 2.033066 | OR725889 |  |  |
| **16** | MAL03 | almogavarii | almogavarii | Mazuby, France | 42.80196 | 2.033066 | OR725890 |  |  |
| **16** | MAL04 | almogavarii | almogavarii | Mazuby, France | 42.80196 | 2.033066 | OR725891 | SRR31341032 |  |
| **16** | MAL05 | almogavarii | almogavarii | Mazuby, France | 42.80196 | 2.033066 | OR725892 | SRR31341031 |  |
| **16** | MAL06 | almogavarii | almogavarii | Mazuby, France | 42.80196 | 2.033066 | OR725893 |  |  |
| **16** | MAL07 | almogavarii | almogavarii | Mazuby, France | 42.80196 | 2.033066 | OR725894 |  |  |
| **17** | ROD01 | almogavarii | almogavarii | Rodome, France | 42.79836 | 2.069658 | OR725917 |  |  |
| **17** | ROD02 | almogavarii | almogavarii | Rodome, France | 42.79836 | 2.069658 | OR725918 |  |  |
| **17** | ROD03 | almogavarii | almogavarii | Rodome, France | 42.79836 | 2.069658 | OR725919 |  |  |
| **17** | ROD04 | almogavarii | almogavarii | Rodome, France | 42.79836 | 2.069658 | OR725920 | SRR31341020 |  |
| **17** | ROD05 | almogavarii | almogavarii | Rodome, France | 42.79836 | 2.069658 | OR725921 |  |  |
| **18** | FONT01 | almogavarii | almogavarii | Fontanès-de-Sault, France | 42.76865 | 2.084398 | OR725868 | SRR31341046 |  |
| **18** | FONT02 | almogavarii | almogavarii | Fontanès-de-Sault, France | 42.76865 | 2.084398 | OR725869 |  |  |
| **18** | FONT03 | almogavarii | almogavarii | Fontanès-de-Sault, France | 42.76865 | 2.084398 | OR725870 |  |  |
| **19** | COU01 | almogavarii | almogavarii | Coudons 1, France | 42.86269 | 2.125448 | OR725847 | SRR31341058 |  |
| **19** | COU02 | almogavarii | almogavarii | Coudons 1, France | 42.86269 | 2.125448 | OR725848 | SRR31341057 |  |
| **19** | COU03 | almogavarii | almogavarii | Coudons 1, France | 42.86269 | 2.125448 | OR725849 |  |  |
| **19** | COU04 | almogavarii | almogavarii | Coudons 1, France | 42.86269 | 2.125448 | OR725850 | SRR31341056 |  |
| **19** | COU05 | almogavarii | almogavarii | Coudons 1, France | 42.86269 | 2.125448 | OR725851 | SRR31341055 |  |
| **19** | COU06 | almogavarii | almogavarii | Coudons 1, France | 42.86269 | 2.125448 | OR725852 |  |  |
| **20*** | JUL01 | almogavarii | almogavarii | Saint-Julia-de-Bec, France | 42.86914 | 2.25695 | OR725791 | SRR31341038 |  |
| **20*** | JUL04 | almogavarii | almogavarii | Saint-Julia-de-Bec, France | 42.86914 | 2.25695 | OR725793 | SRR31341037 |  |
| **20*** | JUL08 | almogavarii | almogavarii | Saint-Julia-de-Bec, France | 42.86914 | 2.25695 | OR725792 | SRR31341036 |  |
| **21*** | FEN02 | almogavarii | almogavarii | Caudiès-de-Fenouillèdes, France | 42.81457 | 2.373498 | OR725861 | SRR31341051 |  |
| **21*** | FEN03 | almogavarii | almogavarii | Caudiès-de-Fenouillèdes, France | 42.81457 | 2.373498 | OR725862 | SRR31341049 |  |
| 22* | SOU01 | almogavarii | almogavarii | Sournia, France | 42.73314 | 2.426531 | OR725944 | SRR31341425 |  |
| 22 | SOU02 | almogavarii | almogavarii | Sournia, France | 42.73314 | 2.426531 | OR725945 | SRR31341424 |  |
| 22 | SOU03 | almogavarii | almogavarii | Sournia, France | 42.73314 | 2.426531 | OR725946 |  |  |
| 22 | SOU04 | almogavarii | almogavarii | Sournia, France | 42.73314 | 2.426531 | OR725947 |  |  |
| 22 | SOU05 | almogavarii | almogavarii | Sournia, France | 42.73314 | 2.426531 | OR725948 |  |  |
| 22 | SOU21_01 | almogavarii |  | Sournia, France | 42.73314 | 2.426531 |  |  | 1 |
| 23* | FI01 | almogavarii | almogavarii | Fillols, France | 42.56088 | 2.409738 | OR725863 | SRR31341048 |  |
| 23* | FI02 | almogavarii | almogavarii | Fillols, France | 42.56088 | 2.409738 | OR725864 | SRR31341047 |  |
| 23 | FI03 | almogavarii | almogavarii | Fillols, France | 42.56088 | 2.409738 | OR725865 |  |  |
| 23 | FI04 | almogavarii | almogavarii | Fillols, France | 42.56088 | 2.409738 | OR725866 |  |  |
| 23 | FI05 | almogavarii | almogavarii | Fillols, France | 42.56088 | 2.409738 | OR725867 |  |  |
| 24* | ALM347 | almogavarii | almogavarii | La Forge-Taulis, France | 42.49858 | 2.657056 | OR725796 | SRR31341083 |  |
| 25* | MNCN81015 | almogavarii | almogavarii | Sant Martin d'Albera, France | 42.46242 | 2.915 | OR725896 | SRR31341030 |  |
| 26* | ALM293 | almogavarii | almogavarii | Sadernes, Spain | 42.2885 | 2.589194 | OR725795 | SRR31341084 |  |
| 27* | ARQ04 | almogavarii | almogavarii | Arques, France | 42.95242 | 2.38355 | OR725797 | SRR31341072 |  |
| 27 | ARQ11 | almogavarii | almogavarii | Arques, France | 42.95242 | 2.38355 | OR725798 |  | 1 |
| 28* | SALZ01 | almogavarii | almogavarii | Salza, France | 42.98343 | 2.496997 | OR725932 | SRR31341436 |  |
| 28* | SALZ02 | almogavarii | almogavarii | Salza, France | 42.98343 | 2.496997 | OR725933 | SRR31341435 |  |
| 28 | SALZ03 | almogavarii | almogavarii | Salza, France | 42.98343 | 2.496997 | OR725934 |  |  |
| 28 | SALZ04 | almogavarii | almogavarii | Salza, France | 42.98343 | 2.496997 | OR725935 |  |  |
| 28 | SALZ05 | almogavarii | almogavarii | Salza, France | 42.98343 | 2.496997 | OR725936 |  |  |
| 29 | GREF11 | almogavarii | almogavarii | Greffeil, France | 43.06876 | 2.374318 | OR725872 |  |  |
| 29* | GREF12 | almogavarii | almogavarii | Greffeil, France | 43.06876 | 2.374318 | OR725873 | SRR31341042 | 1 |
| 29* | GREF13 | almogavarii | almogavarii | Greffeil, France | 43.06876 | 2.374318 | OR725874 | SRR31341041 | 1 |
| 29 | GREF14 | almogavarii | almogavarii | Greffeil, France | 43.06876 | 2.374318 | OR725875 |  | 1 |
| 29 | GREF15 | almogavarii | almogavarii | Greffeil, France | 43.06876 | 2.374318 | OR725876 |  | 1 |
| 30* | COUF01 | almogavarii | almogavarii | Couffoulens, France | 43.15738 | 2.311888 | OR725855 | SRR31341054 |  |
| 30* | COUF02 | almogavarii | almogavarii | Couffoulens, France | 43.15738 | 2.311888 | OR725856 | SRR31341053 |  |
| 30 | COUF03 | almogavarii | almogavarii | Couffoulens, France | 43.15738 | 2.311888 | OR725857 |  |  |
| 30 | COUF04 | almogavarii | almogavarii | Couffoulens, France | 43.15738 | 2.311888 | OR725858 |  |  |
| 30 | COUF05 | almogavarii | almogavarii | Couffoulens, France | 43.15738 | 2.311888 | OR725859 |  |  |
| 31 | BOUG02 | almogavarii | almogavarii | Bouilhounnac, France | 43.24066 | 2.430638 | OR725825 | SRR31341073 |  |
| 31 | BOUG11 | almogavarii |  | Bouilhounnac, France | 43.24066 | 2.430638 |  |  | 1 |
| 32 | VGLY01 | almogavarii | almogavarii | Villegly, France | 43.28379 | 2.442997 | OR725959 |  | 1 |
| 32 | VGLY07 | almogavarii | almogavarii | Villegly, France | 43.28379 | 2.442997 | OR725960 |  |  |
| 32 | VGLY09 | almogavarii | almogavarii | Villegly, France | 43.28379 | 2.442997 | OR725961 |  | 1 |
| 32 | VGLY10 | almogavarii | almogavarii | Villegly, France | 43.28379 | 2.442997 | OR725962 | SRR31341421 | 1 |
| 32 | VGLY11 | almogavarii | almogavarii | Villegly, France | 43.28379 | 2.442997 | OR725963 |  | 1 |
| 32 | VGLY12 | almogavarii |  | Villegly, France | 43.28379 | 2.442997 |  |  | 1 |
| 33 | DOUR01 | obstetricans | obstetricans | Dourgne, France | 43.47702 | 2.147487 | OR725860 | SRR31341052 |  |
| 34* | T3119 | obstetricans | obstetricans | Saint-Etienne-de-Gourgas, France | 43.80901 | 3.37891 | OR725950 | SRR31341423 |  |
| 34* | T3120 | obstetricans | obstetricans | Saint-Etienne-de-Gourgas, France | 43.80901 | 3.37891 | OR725951 | SRR31341422 |  |
| 35* | LAR01 | obstetricans | obstetricans | St-Maurice-de-Navacelles, France | 43.83256 | 3.504816 | OR725885 | SRR31341035 | 1 |
| 35 | LAR02 | obstetricans |  | St-Maurice-de-Navacelles, France | 43.83256 | 3.504816 |  |  | 1 |
| 35* | LAR03 | obstetricans | obstetricans | St-Maurice-de-Navacelles, France | 43.83256 | 3.504816 | OR725886 | SRR31341034 | 1 |
| 35 | LAR04 | obstetricans | obstetricans | St-Maurice-de-Navacelles, France | 43.83256 | 3.504816 | OR725887 |  | 1 |
| 36* | BEV14199 | obstetricans | obstetricans | Crespenou, France | 43.95572 | 3.94513 | OR725820 | SRR31341077 |  |
| 37* | BEV14198 | obstetricans | obstetricans | Courbessac, France | 43.87116 | 4.40037 | OR725819 | SRR31341078 |  |
| 38* | BEV13848 | obstetricans | obstetricans | St-Eulalie, France | 44.81699 | 4.17804 | OR725818 | SRR31341079 |  |
| 39* | JEUR01 | obstetricans | obstetricans | Jeurre, France | 46.36995 | 5.715916 | OR725877 | SRR31341040 |  |
| 39 | JEUR02 | obstetricans | obstetricans | Jeurre, France | 46.36995 | 5.715916 | OR725878 |  |  |
| 39 | JEUR05 | obstetricans |  | Jeurre, France | 46.36995 | 5.715916 |  |  | 1 |
| 39 | JEUR06 | obstetricans |  | Jeurre, France | 46.36995 | 5.715916 |  |  | 1 |
| 40* | R213 | obstetricans | obstetricans | Groeve 't Rooth, Netherlands | 50.838 | 5.775 | OR725910 | SRR31341024 |  |
| 41* | R262 | obstetricans | obstetricans | Keverberg, Netherlands | 50.852 | 5.972 | OR725912 | SRR31341023 |  |
|  | AEB01 | obstetricans | obstetricans | Arrien-en-Bethmale, France | 42.89698 | 1.041388 | OR916157 |  |  |
|  | AEB02 | obstetricans | obstetricans | Arrien-en-Bethmale, France | 42.89698 | 1.041388 | OR916158 |  |  |
|  | AEB03 | obstetricans | obstetricans | Arrien-en-Bethmale, France | 42.89698 | 1.041388 | OR916159 |  |  |
|  | AEB04 | obstetricans | obstetricans | Arrien-en-Bethmale, France | 42.89698 | 1.041388 | OR916160 |  |  |
|  | AEB05 | obstetricans | obstetricans | Arrien-en-Bethmale, France | 42.89698 | 1.041388 | OR916161 |  |  |
|  | AEB06 | obstetricans |  | Arrien-en-Bethmale, France | 42.89698 | 1.041388 |  |  | 1 |
|  | AGO01 | obstetricans | almogavarii | Cadarcet, Fontaine de l'Aigoual, France | 43.01215 | 1.498627 | OR916162 |  |  |
|  | AGO03 | obstetricans | obstetricans | Cadarcet, Fontaine de l'Aigoual, France | 43.01215 | 1.498627 | OR916163 |  |  |
|  | AGO04 | obstetricans | obstetricans | Cadarcet, Fontaine de l'Aigoual, France | 43.01215 | 1.498627 | OR916164 |  |  |
|  | AGO05 | obstetricans |  | Cadarcet, Fontaine de l'Aigoual, France | 43.01215 | 1.498627 |  |  | 1 |
|  | ALM255 | almogavarii | almogavarii | Toses, Spain | 42.31742 | 2.00475 | OR725794 |  |  |
|  | ALYT200523IND1 | obstetricans | obstetricans | Lagnes, France | 43.88618 | 5.11435 | OR916167 |  |  |
|  | ALYT11042023IND1 | almogavarii | almogavarii | Montréal, France | 43.23267 | 2.13415 | OR916149 |  |  |
|  | AL1-01CD11 | almogavarii | almogavarii | Quillan, France | 42.88865 | 2.20047 | OR916150 |  |  |
|  | AL2-01CD66 | almogavarii | almogavarii | Réal, France | 42.63782 | 2.12926 | OR916151 |  |  |
|  | AL3-01CD09 | almogavarii (prob. admixed) | almogavarii | Pamiers, France | 43.11312 | 1.61189 | OR916152 |  |  |
|  | AL3-02CD09 | almogavarii (prob. admixed) | almogavarii | Pamiers, France | 43.11312 | 1.61189 | OR916153 |  |  |
|  | AL4-01CD09 | almogavarii (prob. admixed) | almogavarii | Fougax-et-Barrineuf, France | 42.86267 | 1.91576 | OR916165 |  |  |
|  | AL5-01CD09 | obstetricans | obstetricans | Aulus-les-bains, Etang du Garbet, France | 42.75251 | 1.38004 | OR916166 |  |  |
|  | ALY09OB01 | obstetricans | obstetricans | Montagagne, France | 42.96794 | 1.40709 | OR916154 |  |  |
|  | ALY09OB02 | obstetricans | almogavarii | L’Estagnon, France | 42.80542 | 1.37207 | OR916155 |  |  |
|  | ALY09OB03 | obstetricans | obstetricans | Montesquieu-Avantès, France | 43.03194 | 1.21286 | OR916156 |  |  |
|  | ARV01 | almogavarii (prob. admixed) | almogavarii | Arvigna, France | 43.06459 | 1.74397 | OR725799 |  | 1 |
|  | ARV02 | almogavarii (prob. admixed) | almogavarii | Arvigna, France | 43.06459 | 1.74397 | OR725800 |  |  |
|  | BEV10282 | obstetricans | obstetricans | Saint-Michel 2, France | 43.85342 | 3.389775 | OR725814 |  |  |
|  | BEV10283 | obstetricans | obstetricans | Saint-Michel 2, France | 43.85342 | 3.389775 | OR725815 |  |  |
|  | BEV12047 | obstetricans | obstetricans | Pranlet, France | 44.81328 | 4.26533 | OR725816 |  |  |
|  | BEV13109 | obstetricans | obstetricans | Aumelas, France | 43.60285 | 3.625043 | OR725817 |  |  |
|  | CARBO01 | almogavarii | almogavarii | Lac de Carbonate, France | 43.48123 | 2.256562 | OR725831 |  |  |
|  | CARCAS01 | almogavarii |  | Carcassonne, France | 43.20625 | 2.362223 |  |  | 1 |
|  | COU21_01 | almogavarii | almogavarii | Coudons 2, France | 42.86187 | 2.125124 | OR725853 |  | 1 |
|  | COU21_02 | almogavarii | almogavarii | Coudons 2, France | 42.86187 | 2.125124 | OR725854 |  | 1 |
|  | COU21_03 | almogavarii |  | Coudons 2, France | 42.86187 | 2.125124 |  |  | 1 |
|  | COU21_04 | almogavarii |  | Coudons 2, France | 42.86187 | 2.125124 |  |  | 1 |
|  | COU21_05 | almogavarii |  | Coudons 2, France | 42.86187 | 2.125124 |  |  | 1 |
|  | COU21_06 | almogavarii |  | Coudons 2, France | 42.86187 | 2.125124 |  |  | 1 |
|  | DSA01 | obstetricans | obstetricans | Durban-sur-Arize, France | 43.01689 | 1.339885 | OR916168 |  |  |
|  | DSA02 | obstetricans | obstetricans | Durban-sur-Arize, France | 43.01689 | 1.339885 | OR916169 |  |  |
|  | DSA03 | obstetricans | obstetricans | Durban-sur-Arize, France | 43.01689 | 1.339885 | OR916170 |  | 1 |
|  | DSA04 | obstetricans | obstetricans | Durban-sur-Arize, France | 43.01689 | 1.339885 | OR916171 |  | 1 |
|  | DSA05 | obstetricans | obstetricans | Durban-sur-Arize, France | 43.01689 | 1.339885 | OR916172 |  | 1 |
|  | GAN22_01 | obstetricans |  | Gan, France | 43.22841 | -0.41109 |  |  | 1 |
|  | GAN22_02 | obstetricans |  | Gan, France | 43.22841 | -0.41109 |  |  | 1 |
|  | GAN22_03 | obstetricans |  | Gan, France | 43.22841 | -0.41109 |  |  | 1 |
|  | JO01 | almogavarii | almogavarii | Escaro, France | 42.54123 | 2.302954 | OR725879 |  |  |
|  | JO02 | almogavarii | almogavarii | Escaro, France | 42.54123 | 2.302954 | OR725880 |  |  |
|  | JO03 | almogavarii | almogavarii | Escaro, France | 42.54123 | 2.302954 | OR725881 |  |  |
|  | LAN01 | obstetricans | obstetricans | Lanuéjols, France | 44.14736 | 3.423171 | OR725882 |  |  |
|  | LAN02 | obstetricans | obstetricans | Lanuéjols, France | 44.14736 | 3.423171 | OR725883 |  |  |
|  | LAN03 | obstetricans | obstetricans | Lanuéjols, France | 44.14736 | 3.423171 | OR725884 |  |  |
|  | MD03 | obstetricans | obstetricans | Champsaur, Les Aubins, France | 44.58446 | 6.205152 | OR725895 |  |  |
|  | MD4907 | obstetricans | obstetricans | Champsaur, Les Aubins, France | 44.58446 | 6.205152 | OR916173 |  |  |
|  | MD4909 | obstetricans | obstetricans | Champsaur, Les Aubins, France | 44.58446 | 6.205152 | OR916174 |  |  |
|  | MLV01 | almogavarii (prob. admixed) | almogavarii | Malvieille, France | 42.99983 | 1.71963 | OR916175 |  |  |
|  | MLV02 | almogavarii (prob. admixed) | almogavarii | Malvieille, France | 42.99983 | 1.71963 | OR916176 |  |  |
|  | MLV03 | almogavarii (prob. admixed) | almogavarii | Malvieille, France | 42.99983 | 1.71963 | OR916177 |  |  |
|  | MLV04 | almogavarii (prob. admixed) | almogavarii | Malvieille, France | 42.99983 | 1.71963 | OR916178 |  |  |
|  | MLV05 | almogavarii (prob. admixed) | almogavarii | Malvieille, France | 42.99983 | 1.71963 | OR916179 |  |  |
|  | MOS01 | obstetricans | obstetricans | Saint-Livre, Les Mossières, Switzerland | 46.53463 | 6.3664 | OR725899 |  | 1 |
|  | MOULIS01 | obstetricans |  | Moulis, France | 42.9607 | 1.091897 |  |  | 1 |
|  | PDY01 | almogavarii (prob. admixed) | almogavarii | Pradières, Barry, France | 42.96347 | 1.65314 | OR916180 |  |  |
|  | PDY02 | almogavarii (prob. admixed) | almogavarii | Pradières, Barry, France | 42.96347 | 1.65314 | OR916181 |  |  |
|  | PET01 | almogavarii | almogavarii | La-Part-Petita, France | 42.48541 | 1.965961 | OR725907 |  |  |
|  | PET02 | almogavarii | almogavarii | La-Part-Petita, France | 42.48541 | 1.965961 | OR725908 |  |  |
|  | PET03 | almogavarii | almogavarii | La-Part-Petita, France | 42.48541 | 1.965961 | OR725909 |  |  |
|  | R242 | obstetricans | obstetricans | Kruisbos, Netherlands | 50.803 | 5.913 | OR725911 |  |  |
|  | RLC01 | almogavarii (prob. admixed) | almogavarii | Roquefort-les-cascades, France | 42.95794 | 1.763148 | OR916182 |  |  |
|  | RLC02 | almogavarii (prob. admixed) | almogavarii | Roquefort-les-cascades, France | 42.95794 | 1.763148 | OR916183 |  |  |
|  | RLC03 | almogavarii (prob. admixed) | almogavarii | Roquefort-les-cascades, France | 42.95794 | 1.763148 | OR916184 |  |  |
|  | RLC04 | almogavarii (prob. admixed) | almogavarii | Roquefort-les-cascades, France | 42.95794 | 1.763148 | OR916185 |  |  |
|  | RLC05 | almogavarii (prob. admixed) | almogavarii | Roquefort-les-cascades, France | 42.95794 | 1.763148 | OR916186 |  |  |
|  | RLC06 | almogavarii (prob. admixed) | | Roquefort-les-cascades, France | 42.95794 | 1.763148 |  |  | 1 |
|  | S98 | obstetricans | obstetricans | De Klip 1, Netherlands | 52.157 | 4.379 | OR725930 |  |  |
|  | SALV03 | obstetricans | obstetricans | Le-Salvetat-sur-Agout, France | 43.56664 | 2.738563 | OR725931 |  | 1 |
|  | SALV04 | obstetricans |  | Le-Salvetat-sur-Agout, France | 43.56664 | 2.738563 |  |  | 1 |
|  | SAUV01 | obstetricans | obstetricans | Sauveterre, St-Enimie, France | 44.40895 | 3.440099 | OR725937 |  |  |
|  | SAUV02 | obstetricans | obstetricans | Sauveterre, St-Enimie, France | 44.40895 | 3.440099 | OR725938 |  |  |
|  | T3117 | obstetricans | obstetricans | Saint-Michel 1, France | 43.85394 | 3.404696 | OR725949 |  |  |
|  | T848 | obstetricans | obstetricans | Notre-Dame-de-Londres, France | 43.81458 | 3.78133 | OR725952 |  |  |
|  | T849 | obstetricans | obstetricans | Notre-Dame-de-Londres, France | 43.81458 | 3.78133 | OR725953 |  |  |
|  | T850 | obstetricans | obstetricans | Notre-Dame-de-Londres, France | 43.81458 | 3.78133 | OR725954 |  |  |
|  | TJ01 | almogavarii | almogavarii | Enveitg, France | 42.45902 | 1.905824 | OR725955 |  |  |
|  | TJ02 | almogavarii | almogavarii | Enveitg, France | 42.45902 | 1.905824 | OR725956 |  |  |
|  | TJ03 | almogavarii | almogavarii | Enveitg, France | 42.45902 | 1.905824 | OR725957 |  |  |
|  | TJ08 | almogavarii | almogavarii | Enveitg, France | 42.45902 | 1.905824 | OR725958 |  |  |
|  |  |  |  |  |  |  |  |  |  |

^1^ https://doi.org/10.5281/zenodo.10037218
