# Supplementary material for: Genomic and bioacoustic variation in a midwife toad hybrid zone: A role for reinforcement?
Source: PLoS One. 2024 Nov 25;19(11):e0314477. doi: 10.1371/journal.pone.0314477 (PMC11588267; doi:10.1371/journal.pone.0314477)
Supplement: S2 Table — (DOCX) [file pone.0314477.s002.docx]

**S2 Table. Numbers of individuals with the *A. o. obstetricans* and *A. a. almogavarii* mtDNA lineages in genetically barcoded localities, combining our study and the literature.**

| **Locality** | **Y** | **X** | ***obstetricans*** | ***almogavarii*** | **Source** |
| --- | --- | --- | --- | --- | --- |
| Sournia | 42.73314 | 2.426531 | 0 | 5 | 1 |
| Fillols | 42.56088 | 2.409738 | 0 | 5 | 1 |
| Escaro | 42.54123 | 2.302954 | 0 | 3 | 1 |
| La-Part-Petita | 42.48541 | 1.965961 | 0 | 3 | 1 |
| Enveitg | 42.45902 | 1.905824 | 0 | 4 | 1 |
| Perles | 42.74419 | 1.786684 | 0 | 7 | 1 |
| Saint-Cirac | 42.94161 | 1.70461 | 0 | 9 | 1 |
| Roquefixade | 42.93553 | 1.755277 | 0 | 8 | 1 |
| Montels | 43.00461 | 1.472231 | 10 | 0 | 1 |
| Samortein | 42.88917 | 1.05645 | 3 | 0 | 1 |
| Coudons 1 | 42.86269 | 2.125448 | 0 | 6 | 1 |
| Coudons 2 | 42.86187 | 2.125124 | 0 | 2 | 1 |
| Mazuby | 42.80196 | 2.033066 | 0 | 7 | 1 |
| Rodome | 42.79836 | 2.069658 | 0 | 5 | 1 |
| Fontanès-de-Sault | 42.76865 | 2.084398 | 0 | 3 | 1 |
| Saleich | 43.03515 | 0.965823 | 6 | 0 | 1 |
| Avezac | 43.06645 | 0.337807 | 3 | 0 | 1 |
| Mont-de-Galié | 42.98972 | 0.647247 | 1 | 0 | 1 |
| St-Maurice-de-Navacelles | 43.83256 | 3.504816 | 3 | 0 | 1 |
| Le-Salvetat-sur-Agout | 43.56664 | 2.738563 | 1 | 0 | 1 |
| Villegly | 43.28379 | 2.442997 | 0 | 5 | 1 |
| Bouilhounnac | 43.24066 | 2.430638 | 0 | 1 | 1 |
| Couffoulens | 43.15738 | 2.311888 | 0 | 5 | 1 |
| Salza | 42.98343 | 2.496997 | 0 | 5 | 1 |
| Greffeil | 43.06876 | 2.374318 | 0 | 5 | 1 |
| Arques | 42.95242 | 2.38355 | 0 | 2 | 1 |
| Saint-Julia-de-Bec | 42.86914 | 2.25695 | 0 | 3 | 1 |
| Caudiès-de-Fenouillèdes | 42.81457 | 2.373498 | 0 | 2 | 1 |
| Saint-Etienne-de-Gourgas | 43.80901 | 3.37891 | 2 | 0 | 1 |
| Pranlet | 44.81328 | 4.26533 | 1 | 0 | 1 |
| St-Eulalie | 44.81699 | 4.17804 | 1 | 0 | 1 |
| Courbessac | 43.87116 | 4.40037 | 1 | 0 | 1 |
| Crespenou | 43.95572 | 3.94513 | 1 | 0 | 1 |
| Etang de Lers | 42.80731 | 1.382611 | 2 | 0 | 1 |
| Sant Martin d'Albera | 42.46242 | 2.915 | 0 | 1 | 1 |
| La Forge-Taulis | 42.49858 | 2.657056 | 0 | 1 | 1 |
| Sadernes | 42.2885 | 2.589194 | 0 | 1 | 1 |
| Toses | 42.31742 | 2.00475 | 0 | 1 | 1 |
| Saint-Michel2 | 43.85342 | 3.389775 | 2 | 0 | 1 |
| Aumelas2 | 43.60285 | 3.625043 | 1 | 0 | 1 |
| Notre-Dame-de-Londres 1 | 43.81458 | 3.78133 | 3 | 0 | 1 |
| Saint-Michel 1 | 43.85394 | 3.404696 | 1 | 0 | 1 |
| Arvigna | 43.06459 | 1.743967 | 0 | 2 | 1 |
| Becq | 42.94497 | 1.57891 | 3 | 1 | 1 |
| Benaix | 42.90614 | 1.85062 | 0 | 3 | 1 |
| Ganac | 42.94792 | 1.558236 | 4 | 0 | 1 |
| Caraybat | 42.94187 | 1.671247 | 0 | 5 | 1 |
| Lac de Carbonate | 43.48123 | 2.256562 | 0 | 1 | 1 |
| Dourgne | 43.47702 | 2.147487 | 1 | 0 | 1 |
| Jeurre | 46.36995 | 5.715916 | 2 | 0 | 1 |
| Lanuéjols | 44.14736 | 3.423171 | 3 | 0 | 1 |
| Champsaur, Les Aubins | 44.58446 | 6.205152 | 3 | 0 | 1 |
| Saint-Livre, Les Mossières | 46.53463 | 6.3664 | 1 | 0 | 1 |
| Recort | 42.95737 | 1.538699 | 1 | 3 | 1 |
| Sauveterre, St-Enimie | 44.40895 | 3.440099 | 2 | 0 | 1 |
| Sentenac-de-Sérou | 42.97258 | 1.388478 | 5 | 0 | 1 |
| Cadarcet, fontaine de l'Aigoual | 43.01215 | 1.498627 | 2 | 1 | 1 |
| Arrien-en-Bethmale | 42.89698 | 1.041388 | 5 | 0 | 1 |
| Durban-sur-Arize | 43.01689 | 1.339885 | 5 | 0 | 1 |
| Roquefort-les-cascades | 42.95794 | 1.763148 | 0 | 5 | 1 |
| Malvieille | 42.99983 | 1.719627 | 0 | 5 | 1 |
| Pradières, Barry | 42.96347 | 1.653138 | 0 | 2 | 1 |
| Lagnes | 43.88618 | 5.114345 | 1 | 0 | 1 |
| Fougax-et-Barrineuf | 42.86267 | 1.915764 | 0 | 1 | 1 |
| Aulus-les-Bains, Etg. du Garbet | 42.75251 | 1.38004 | 1 | 0 | 1 |
| Montréal | 43.23267 | 2.134152 | 0 | 1 | 1 |
| Quillan | 42.88865 | 2.200471 | 0 | 1 | 1 |
| Réal | 42.63782 | 2.129261 | 0 | 1 | 1 |
| Pamiers | 43.11312 | 1.611889 | 0 | 2 | 1 |
| Montagagne | 42.96794 | 1.407085 | 1 | 0 | 1 |
| L'estagnon | 42.80542 | 1.372071 | 0 | 1 | 1 |
| Montesquieu-Avantès | 43.03194 | 1.212861 | 1 | 0 | 1 |
| Castellbisbal | 41.4943 | 1.9385 | 0 | 3 | 2 |
| Figuerola del Camp | 41.3752 | 1.2626 | 0 | 2 | 2 |
| Garraf | 41.2736 | 1.837 | 0 | 10 | 2 |
| Mediona | 41.473 | 1.639 | 0 | 6 | 2 |
| Mura | 41.6956 | 1.9855 | 0 | 5 | 2 |
| Pontils | 41.4811 | 1.3748 | 0 | 10 | 2 |
| Pontons | 41.4215 | 1.5159 | 0 | 6 | 2 |
| San Joan de Mediona | 41.4618 | 1.5902 | 0 | 4 | 2 |
| Sant Llorenç del Munt | 41.6507 | 1.9821 | 0 | 5 | 2 |
| St Magi de Brufaganya | 41.4851 | 1.44 | 0 | 3 | 2 |
| Vilaperdius | 41.4792 | 1.4086 | 0 | 3 | 2 |
| A Coruña | 43.375 | -8.433 | 1 | 0 | 3 |
| Alfonxe | 42.924 | -7.389 | 2 | 0 | 3 |
| Casavegas | 43.022 | -4.513 | 1 | 0 | 3 |
| El Fito | 43.433 | -5.148 | 1 | 0 | 3 |
| El Port d’Armentera | 41.387 | 1.359 | 0 | 6 | 2, 3 |
| Fonchanina | 42.522 | 0.652 | 0 | 1 | 3 |
| Fresnedo 1 | 43.367 | -3.567 | 1 | 0 | 3 |
| Fresnedo 2 | 43.35 | -3.333 | 3 | 0 | 3 |
| Irati | 42.993 | -1.1 | 2 | 0 | 3 |
| Iraty | 43.046 | -1.074 | 1 | 0 | 3 |
| Jublains | 48.251 | -0.501 | 2 | 0 | 3 |
| La Bisbal | 41.939 | 2.949 | 0 | 2 | 3 |
| La Coma | 42.188 | 1.581 | 0 | 4 | 3 |
| La Sallète | 43.993 | 2.167 | 1 | 0 | 3 |
| Lago de Isoba | 43.046 | -5.315 | 3 | 0 | 3 |
| Llinars | 42.131 | 1.709 | 0 | 10 | 3 |
| Piera | 41.511 | 1.737 | 0 | 11 | 2, 3 |
| Prades | 41.795 | 1.579 | 0 | 2 | 3 |
| Puerto de San Isidro | 43.05 | -5.325 | 1 | 0 | 3 |
| Rasos de Peguera | 42.136 | 1.761 | 0 | 6 | 3 |
| Rhür | 51.517 | 7.45 | 1 | 0 | 3 |
| Ribaforada | 42.006 | -1.536 | 1 | 0 | 3 |
| Riudarenes | 41.822 | 2.717 | 0 | 1 | 3 |
| Saint-Michel | 43.168 | 1.084 | 1 | 0 | 3 |
| Sant Hilari Sacalm | 41.903 | 2.506 | 0 | 2 | 3 |
| Sant Miquel del Fai | 41.716 | 2.193 | 0 | 7 | 2, 3 |
| Sierra de Andía | 42.818 | -1.97 | 1 | 0 | 3 |
| Soldes, Túnel del Cadí | 42.35 | 1.7 | 0 | 2 | 3 |
| Somiedo | 43.07 | -6.306 | 2 | 0 | 3 |
| Tineo | 43.333 | -6.417 | 1 | 0 | 3 |
| Tolivia | 43.2 | -5.583 | 1 | 0 | 3 |
| Tudanca | 43.15 | -4.367 | 3 | 0 | 3 |
| Veguellina de Órbigo | 42.45 | -5.885 | 1 | 0 | 3 |
| Vizcaya | 43.088 | -2.792 | 2 | 0 | 3 |
| Tuchan | 42.885 | 2.733 | 0 | 1 | 3 |
| Abeurador Abella | 42.452 | 0.564 | 0 | 1 | 4 |
| Abeurador Boscalt | 42.314 | 1.587 | 0 | 2 | 4 |
| Abeurador Sarroqueta | 42.444 | 0.724 | 0 | 2 | 4 |
| Abeurador Señuy | 42.46 | 0.641 | 0 | 2 | 4 |
| Ándara | 43.213 | -4.716 | 1 | 0 | 4 |
| Balsa Pertacua | 42.714 | -0.423 | 1 | 0 | 4 |
| Barranc de Viu | 42.373 | 0.812 | 0 | 2 | 4 |
| Barranco Las Foyas | 42.862 | -0.696 | 2 | 0 | 4 |
| Barranco Llana de Obarra | 42.528 | 0.653 | 0 | 2 | 4 |
| Bassa d'Arres | 42.769 | 0.715 | 4 | 0 | 4 |
| Bassa Manyanet | 42.47 | 0.908 | 0 | 1 | 4 |
| Bassa Vallibierna | 42.613 | 0.61 | 0 | 3 | 4 |
| Bellver de Cerdanya | 42.372 | 1.777 | 0 | 1 | 4 |
| Canfranc | 42.717 | -0.523 | 1 | 0 | 4 |
| Castellterçol | 41.763 | 2.118 | 0 | 1 | 4 |
| Enveitg | 42.459 | 1.906 | 0 | 3 | 4 |
| Estanhet d'Arcoïls | 42.68 | 0.988 | 3 | 0 | 4 |
| Estanho Vilac | 42.709 | 0.814 | 2 | 0 | 4 |
| Estany Basibé | 42.548 | 0.596 | 0 | 2 | 4 |
| Estany d'Aulà | 42.769 | 1.099 | 3 | 0 | 4 |
| Estany de Clavera | 42.778 | 1.077 | 3 | 0 | 4 |
| Estanyet Coma d'Espós | 42.509 | 1.017 | 0 | 1 | 4 |
| Estanyet de Davall | 42.415 | 1.232 | 0 | 3 | 4 |
| Ferran | 41.73 | 1.401 | 0 | 1 | 4 |
| Formigal | 42.775 | -0.376 | 3 | 0 | 4 |
| Fte. Nueva de Bardales | 41.424 | -5.344 | 2 | 0 | 4 |
| Gavet de la Conca | 42.045 | 1.037 | 0 | 2 | 4 |
| Ibón Acherito | 42.88 | -0.707 | 5 | 0 | 4 |
| Ibón Campo de Troya Inferior | 42.766 | -0.409 | 3 | 0 | 4 |
| Ibón de los Asnos | 42.693 | -0.267 | 2 | 0 | 4 |
| Ibón Espelunciecha | 42.787 | -0.431 | 1 | 0 | 4 |
| Ibón Negras | 42.787 | -0.465 | 4 | 0 | 4 |
| Ibón Orná | 42.798 | -0.614 | 3 | 0 | 4 |
| Ibón Serrato Alto | 42.765 | -0.213 | 4 | 0 | 4 |
| Ibón Serrato Bajo | 42.765 | -0.215 | 2 | 0 | 4 |
| Ibón Viejo | 42.781 | -0.602 | 2 | 0 | 4 |
| Isaba | 42.946 | -0.835 | 2 | 0 | 4 |
| La Goda | 41.563 | 1.446 | 0 | 2 | 4 |
| La Pobla de Segur | 42.25 | 0.963 | 0 | 1 | 4 |
| Lac d'Arlet | 42.839 | -0.615 | 2 | 0 | 4 |
| Lac de Lhurs | 42.922 | -0.704 | 2 | 0 | 4 |
| Lac du Saut de Vésoles | 43.555 | 2.794 | 1 | 0 | 4 |
| Lago de Valdominguero | 43.207 | -4.727 | 3 | 0 | 4 |
| Lago Ercina | 43.267 | -4.979 | 2 | 0 | 4 |
| Les Paüls | 42.408 | 0.606 | 0 | 1 | 4 |
| Lescun | 42.934 | -0.637 | 3 | 0 | 4 |
| Liordes | 43.149 | -4.857 | 3 | 0 | 4 |
| Llagos de Jesús | 43.175 | -5.056 | 2 | 0 | 4 |
| Murua | 42.976 | -2.736 | 1 | 0 | 4 |
| Naval Aguas Tuertas | 42.812 | -0.621 | 3 | 0 | 4 |
| Navès | 42.088 | 1.677 | 0 | 1 | 4 |
| Noves de Segre | 42.295 | 1.342 | 0 | 2 | 4 |
| Pilón de Igüedri | 43.146 | -4.774 | 3 | 0 | 4 |
| Pilón de Moñetas | 43.202 | -4.783 | 3 | 0 | 4 |
| Pilón de Pandébano | 43.235 | -4.781 | 3 | 0 | 4 |
| Pilón Vegas de Sotres | 43.208 | -4.767 | 3 | 0 | 4 |
| Pilón Vegas del Enol | 43.269 | -4.998 | 3 | 0 | 4 |
| Pla de Beret | 42.725 | 0.964 | 1 | 0 | 4 |
| Plano de Igüer | 42.745 | -0.588 | 1 | 0 | 4 |
| Pobla de Carivenys | 41.57 | 1.441 | 0 | 2 | 4 |
| Pozo de Moñetas | 43.197 | -4.786 | 3 | 0 | 4 |
| Pozos de Lloroza | 43.165 | -4.811 | 2 | 0 | 4 |
| Puerto de Lizarraga | 42.86 | -2.005 | 1 | 0 | 4 |
| Puit d'Arious | 42.864 | -0.633 | 2 | 0 | 4 |
| Serra de l’Orri | 42.428 | 1.205 | 0 | 1 | 4 |
| Tramacastilla de Tena | 42.705 | -0.32 | 1 | 0 | 4 |
| Valle del Tendi | 43.302 | -5.249 | 1 | 0 | 4 |
| St Pierre-de-la-Fage | 43.794 | 3.42 | 1 | 0 | 5 |
| Eckelrade | 50.823 | 5.764 | 4 | 0 | 5 |
| Mheer | 50.774 | 5.78 | 4 | 0 | 5 |
| Noordbeek | 50.77 | 5.826 | 4 | 0 | 5 |
| Groeve Blom | 50.857 | 5.792 | 4 | 0 | 5 |
| Meertensgroeve | 50.862 | 5.804 | 4 | 0 | 5 |
| Curfsgroeve | 50.872 | 5.76 | 4 | 0 | 5 |
| Kloosterbosch-Meerssen | 50.878 | 5.796 | 4 | 0 | 5 |
| Emmaberg | 50.875 | 5.848 | 3 | 0 | 5 |
| Schaelsberg | 50.86 | 5.857 | 2 | 0 | 5 |
| Bemelerberg | 50.85 | 5.765 | 4 | 0 | 5 |
| Julianagroeve | 50.832 | 5.778 | 4 | 0 | 5 |
| Kunderberg | 50.864 | 5.95 | 4 | 0 | 5 |
| Putberg | 50.855 | 5.966 | 4 | 0 | 5 |
| Ubachsberg zuid | 50.856 | 5.96 | 4 | 0 | 5 |
| Holset | 50.775 | 5.988 | 4 | 0 | 5 |
| Langenberg Goslar | 51.903 | 10.508 | 4 | 0 | 5 |
| Velbert | 51.34 | 7.047 | 4 | 0 | 5 |
| De Klip 1 | 52.157 | 4.379 | 11 | 0 | 1, 5 |
| De Klip 2 | 52.157 | 4.378 | 2 | 0 | 5 |
| Den Haag 1 | 52.079 | 4.247 | 2 | 0 | 5 |
| Den Haag 2 | 52.1 | 4.278 | 1 | 0 | 5 |
| Den Haag 3 | 52.1 | 4.284 | 2 | 0 | 5 |
| Den Haag 4 | 52.07924 | 4.24734 | 1 | 0 | 5 |
| Den Haag 5 | 52.09957 | 4.27879 | 1 | 0 | 5 |
| Groot-Loon | 50.794 | 5.362 | 2 | 0 | 5 |
| Voeren - Veurs | 50.736 | 5.851 | 2 | 0 | 5 |
| St Genesius Rode | 50.732 | 4.362 | 2 | 0 | 5 |
| Terril St Antoine | 50.413 | 3.772 | 2 | 0 | 5 |
| Grande Honnelle | 50.342 | 3.726 | 2 | 0 | 5 |
| Camp militaire de Marche en Famenne | 50.29 | 5.371 | 2 | 0 | 5 |
| Carrière de Stockay | 50.577 | 5.379 | 2 | 0 | 5 |
| Carrière de Marchempré | 50.476 | 5.022 | 1 | 0 | 5 |
| Voeren - Kattenroth | 50.757 | 5.802 | 1 | 0 | 5 |
| Kruisbos | 50.803 | 5.913 | 4 | 0 | 1, 5 |
| Keverberg | 50.852 | 5.972 | 4 | 0 | 1, 5 |
| Groeve 't Rooth | 50.838 | 5.775 | 5 | 0 | 1, 5 |
| Villeneuve-Minervois | 43.3239 | 2.4556 | 0 | 1 | 6 |
|  |  |  |  |  |  |

^1^ this study; ^2^ ref. [53]; ^3^ ref. [54]; ^4^ ref. [57] ; ^5^ ref. [58] ; ^6^ P-A Crochet pers. comm.
