# Supplementary material for: Genomic and bioacoustic variation in a midwife toad hybrid zone: A role for reinforcement?
Source: PLoS One. 2024 Nov 25;19(11):e0314477. doi: 10.1371/journal.pone.0314477 (PMC11588267; doi:10.1371/journal.pone.0314477)
Supplement: S3 Table — For mtDNA and Q, numbers correspond to the averages and 95% confidence intervals. For the species-diagnostic loci, numbers correspond to the median and standard deviation among the 4,092 SNPs. (DOCX) [file pone.0314477.s003.docx]

**S3 Table. Cline parameters *c* (center) and *w* (width) for the different datasets.** For mtDNA and *Q*, numbers correspond to the averages and 95% confidence intervals. For the species-diagnostic loci, numbers correspond to the median and standard deviation among the 4,092 SNPs.

|  | **individuals** | **localities** | ***c*** | ***w*** |
| --- | --- | --- | --- | --- |
|  |  |  |  |  |
| **mtDNA** | 93 | 19 | 101.7 (98.9–104.3) | 9.8 (6.4–17.1) |
| **Genome average (*Q*)** | 58 | 19 | 106.5 (103.2–111.4) | 20.6 (12.5–41.4) |
| **Species-diagnostic loci** | 57 | 19 | 106.9 ± 4.0 | 21.2 ± 9.5 |
|  |  |  |  |  |
|  |  |  |  |  |
