# Supplementary material for: Genomic and bioacoustic variation in a midwife toad hybrid zone: A role for reinforcement?
Source: PLoS One. 2024 Nov 25;19(11):e0314477. doi: 10.1371/journal.pone.0314477 (PMC11588267; doi:10.1371/journal.pone.0314477)
Supplement: S4 Table — DF: dominant frequency; ND: note duration; RT: rising time; PR: pulse rate. (DOCX) [file pone.0314477.s004.docx]

**S4 Table. Mean, standard variation (±) and range (in brackets) of the bioacoustic variables in the four groups.** DF: dominant frequency; ND: note duration; RT: rising time; PR: pulse rate.

|  | individuals | notes | DF (Hz) | ND (s) | RT (s) | PR (s^-1^) |
| --- | --- | --- | --- | --- | --- | --- |
|  |  |  |  |  |  |  |
| *obstetricans* allopatric | 27 | 156 | 1319.6 ± 133.1 (1119.7-1636.5) | 0.094 ± 0.020 (0.065-0.152) | 0.008 ± 0.002 (0.003-0.016) | 1309 ± 133 (1111-1663) |
| *almogavarii* allopatric | 22 | 120 | 1431.9 ± 114.4 (1206.0-1636.5) | 0.090 ± 0.014 (0.071-0.144) | 0.009 ± 0.003 (0.001-0.019) | 1412 ± 121 (1186-1615) |
| *obstetricans* parapatric | 9 | 52 | 1261.4 ± 41.2 (1205.8-1335.1) | 0.084 ± 0.012 (0.069-0.111) | 0.011 ± 0.004 (0.004-0.019) | 1259 ± 39 (1198-1345) |
| *almogavarii* parapatric | 13 | 78 | 1354.9 ± 79.3 (1248.9-1507.3) | 0.086 ± 0.009 (0.064-0.109) | 0.008 ± 0.003 (0.005-0.022) | 1353 ± 78 (1253-1507) |
|  |  |  |  |  |  |  |
|  |  |  |  |  |  |  |
