# Supplementary material for: Genomic and bioacoustic variation in a midwife toad hybrid zone: A role for reinforcement?
Source: PLoS One. 2024 Nov 25;19(11):e0314477. doi: 10.1371/journal.pone.0314477 (PMC11588267; doi:10.1371/journal.pone.0314477)

**S1 Fig. Oscillogram (top) and spectrogram (bottom) of a note, showing the variables measured.** PR is obtained by counting the number of P pulses per time unit (dividing by ND).


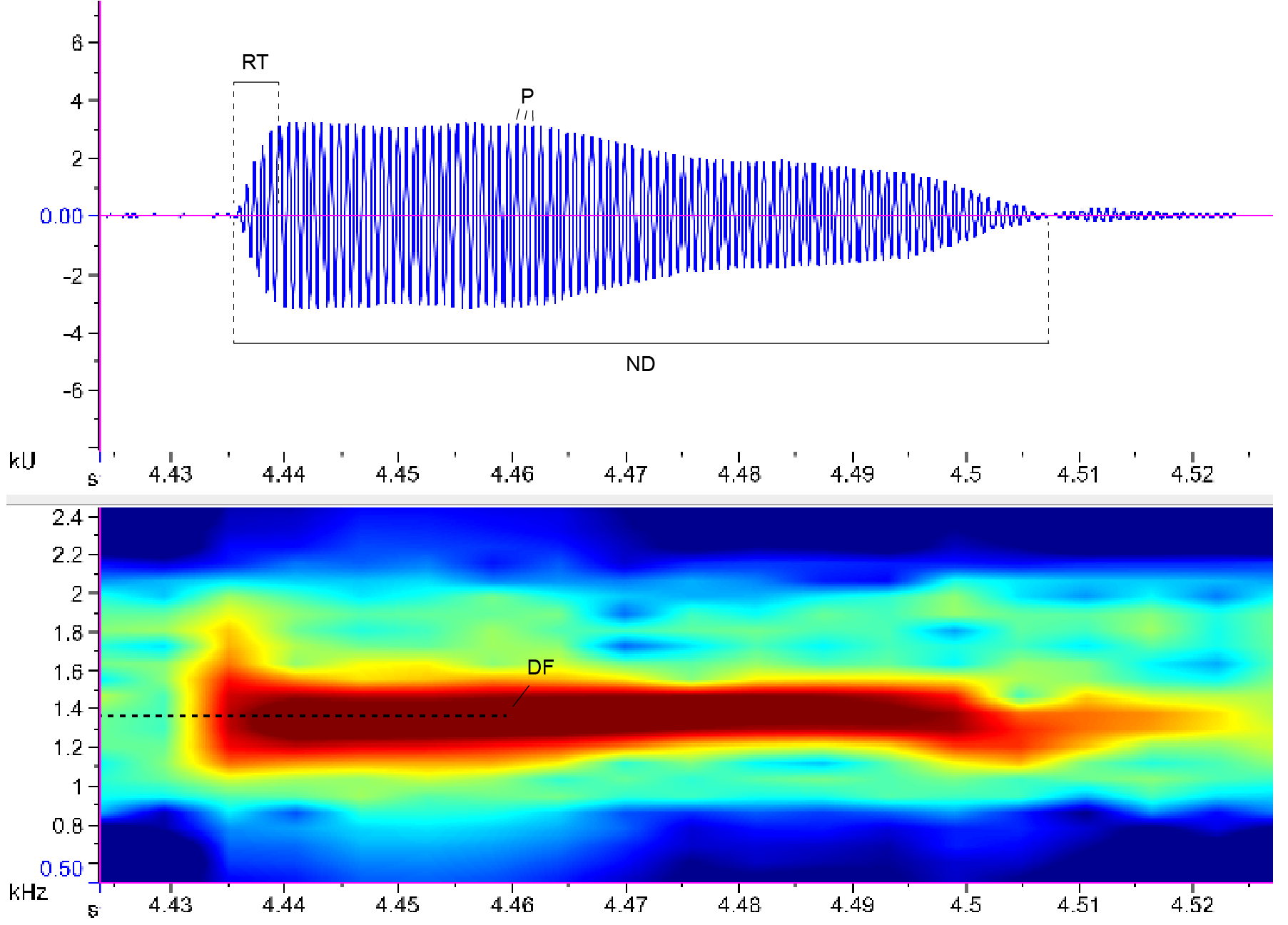

Supplement: S1 Fig — Oscillogram (top) and spectrogram (bottom) of a note, showing the variables measured. PR is obtained by counting the number of P pulses per time unit (dividing by ND). (DOCX) [file pone.0314477.s005.docx]
