# Supplementary figures and images for: Genomic and bioacoustic variation in a midwife toad hybrid zone: A role for reinforcement?
Source: PLoS One. 2024 Nov 25;19(11):e0314477. doi: 10.1371/journal.pone.0314477 (PMC11588267; doi:10.1371/journal.pone.0314477)

**S2 Fig. ML phylogenetic analysis of the 16S sequences for DNA barcoding.**


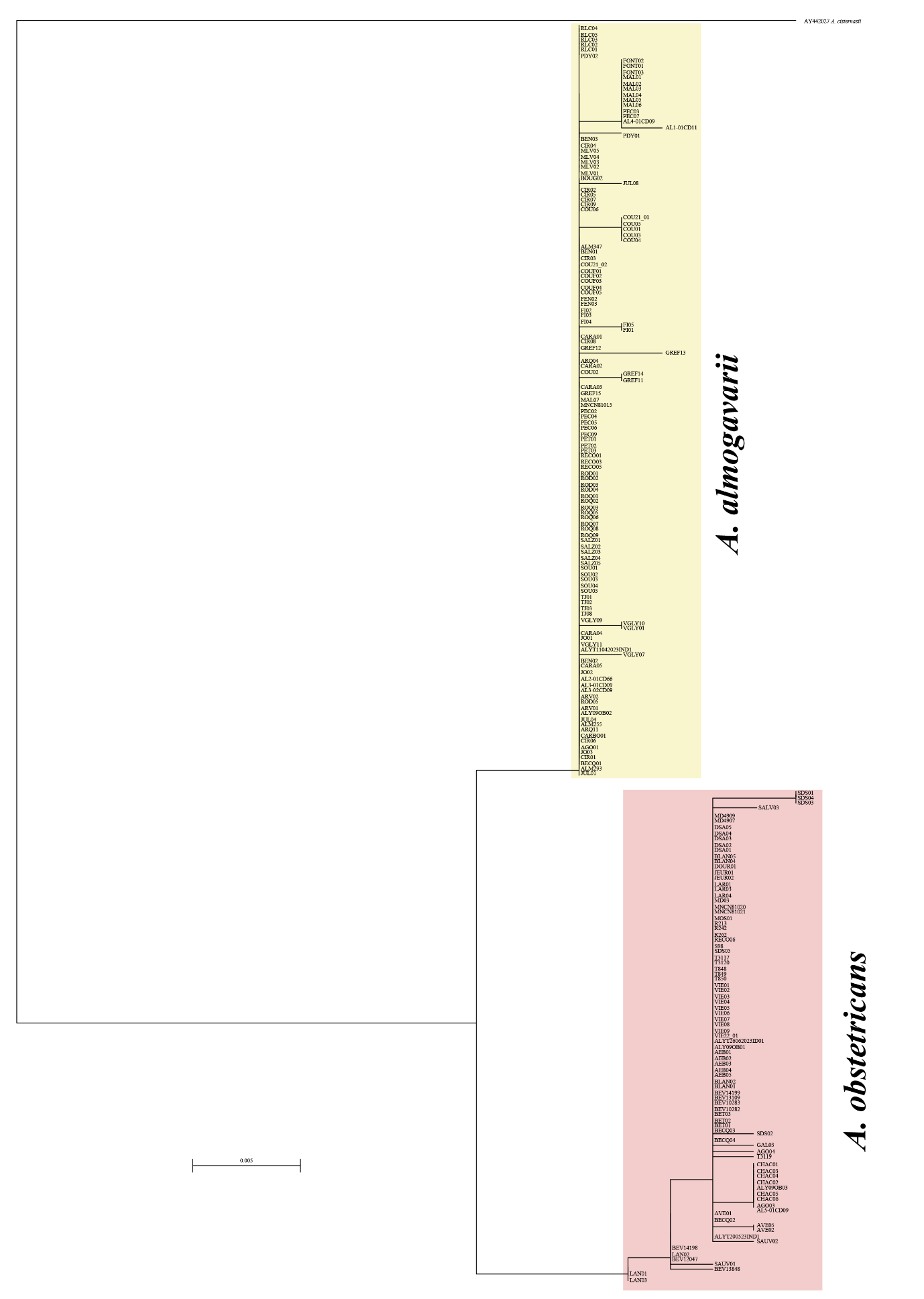

Supplement: S2 Fig — (DOCX) [file pone.0314477.s006.docx]
