# Supplementary material for: Genomic and bioacoustic variation in a midwife toad hybrid zone: A role for reinforcement?
Source: PLoS One. 2024 Nov 25;19(11):e0314477. doi: 10.1371/journal.pone.0314477 (PMC11588267; doi:10.1371/journal.pone.0314477)
Supplement: Attachment — Submitted filename: Draft_PLoS_ONE_revised.docx [file pone.0314477.s007.docx]

**Genomic and bioacoustic variation in a midwife toad hybrid zone: a role for reinforcement?**

Johanna Ambu^1^, Christophe Dufresnes^1,2^

^1^ Laboratory for Amphibian Systematics and Evolution, College of Biology & the Environment, Nanjing Forestry University, Nanjing, China.

^2^ Institut de Systématique, Evolution, Biodiversité (ISYEB), Muséum national d’Histoire naturelle, CNRS, Sorbonne Université, EPHE, Université des Antilles, Paris, France.

**Corresponding author:** Christophe Dufresnes; [Christophe.Dufresnes@hotmail.fr](mailto:Christophe.Dufresnes@hotmail.fr)

**Abstract**

Hybrid zones, i.e., geographic areas where diverging lineages meet, hybridize and eventually mix their genomes, offer opportunities to understand the mechanisms behind reproductive isolation and speciation. Hybrid zones are particularly well suited to study reinforcement, i.e., the process by which selection against hybridization increases reproductive barriers, which, in anuran amphibians, is typically expressed by increased divergence in advertisement calls – the main cue to assortative mating – in parapatric ranges. Using mitochondrial barcoding (16S sequences), population genomics (thousands of SNPs) and bioacoustic analyses (four call parameters), we examine the hybrid zone between two incipient species of midwife toads (*Alytes obstetricans* and *A. almogavarii*) in southern France, with the purposes of locating their transition, measuring genetic introgression, and documenting potential signatures of reinforcement. We map range boundaries in the Eastern Pyrenees and the southwestern foothills of the Massif Central, namely along the Ariège valley and the Montagne Noire area. Similarly to another transition between these species in Spain, we found the hybrid zone to be narrow, involving geographically restricted gene flow (~20 km wide allele frequency clines) and barrier loci (i.e., loci resisting introgression), both suggestive of partial post-zygotic isolation (hybrid incompatibilities). The calls of the species overlap less inside than outside the hybrid zone, due to a reduction of their standing variation rather than a shift towards distinctive variants. While neutral causes cannot be excluded, this pattern follows the general expectations of reinforcement, yet without reproductive character displacement. Our study highlights the potential of amphibian hybrid zones to assess the genetic and behavioral drivers of reproductive isolation *in statu nascendi* and under various evolutionary contexts.

**Keywords**

*Alytes*; Hybridization; Reproductive Character Displacement; Reproductive Isolation; Speciation

**Introduction**

Reinforcement, i.e., the process by which reproductive barriers between incipient species are strengthened by natural selection, is one of the most discussed mechanisms of speciation (Dobzhansky 1937, 1940, Butlin 1987, Howard 1993, Coyne & Orr 2004, Lukhtanov 2011). Reinforcement typically acts in secondary contact zones and involves an increase of phenotypic differentiation between species that enhances their ability to avoid and/or discriminate each other via pre-mating barriers, and thus escape the fitness costs associated with maladaptive hybridization. Reinforcement has found support among ubiquitous taxa, including vertebrates (e.g., mammals, Smadja & Ganem 2005; birds, Saetre et al. 1997, Vallin et al. 2012, Sebastianelli 2023; fishes, Rundle & Schluter 1998, Baiz et al. 2019, St John & Feller 2021; amphibians, Pfennig & Simovich 2002; Pfennig 2003, Pfennig & Rice 2014), invertebrates (e.g., insects, Coyne & Orr 1989, Noor 1995, Hostert 1997, Higgie et al. 2000, Nosil et al. 2003, Ortiz-Barrientos et al. 2004, Peterson et al. 2005, Kronforst et al. 2007, Souza et al. 2008, Porretta & Urbanelli 2012, Yukilevich 2012, 2021), and plants (Ramsey et al. 2003, Silvertown et al. 2005, Kay & Schmeske 2008, Pellegrino 2016, Roda et al. 2017). Although reinforcement has received ample empirical and theoretical consideration (e.g., Servedio & Noor 2003, Coyne & Orr 2004, Lemmon et al. 2004, Hopkins 2013, Butlin & Smadja, 2018), its incidence and mode of operation remain elusive in many organisms.

Reinforcement in animals has been preferentially measured by comparing inter-specific behavioral differences in areas of sympatry *vs* areas of allopatry, in the form of reproductive character displacement, i.e., a disruptive shift in the traits involved in mate recognition (Fig. 1, left). The signal may be complex, involving sex-specific and species-specific asymmetries, depending on a variety of factors such as the reproductive investment of females compared to males, the differential fitness of reciprocal cross directions, or the relative population sizes of the interacting species (Servedio & Noor 2003; Coyne & Orr 2004, Yukilevich 2012). Alternatively, species differences may be reached without displacement (Fig. 1, right). For polymorphic species, non-overlapping reproductive traits can be achieved by reducing the existing variation rather than evolving towards new phenotypes (Servedio & Noor 2003, Lemmon et al. 2004).

Amphibians have long been considered a promising model to study reinforcement (Blair 1964, Butlin 1987) and some seminal work has demonstrated reproductive character displacements on coloration (e.g., poison frogs, Twomey et al. 2014; *Lissotriton* newts, de Solan et al. 2022) and advertisement calls (e.g., North American Chorus frogs, Lemmon 2009, Lemmon & Juenger 2017; Australian tree frogs, Hoskin et al. 2005). In anurans (frogs and toads), many species use vocalizations for mate recognition (Fouquette 1960, Köhler et al. 2017), sometimes with high intra- and inter-specific variation (Weaver et al. 2020). Most of this variation is under genetic control and thus heritable (Kime et al. 2000, Hoffmann & Reyer 2013). In parallel, many anuran lineages meet in secondary contact zones from where to characterize the onset of genetic and behavioral reproductive barriers in concert (Hewitt 1998).

Here we examine genetic and bioacoustic variation in a hybrid zone between two incipient species of midwife toads, *Alytes obstetricans* and *A. almogavarii*. In midwife toads, mate recognition relies on loud high-pitched vocalizations, and males invest as much reproductive effort as females due to a remarkable behavior of parental care that consists of nursing the eggs until they hatch (Lange et al. 2022). The species under focus belong to a species complex that diversified in Western Europe within the last 4 My, with six phylogeographic lineages that retain high phenotypic (notably bioacoustic) polymorphism and meet in multiple contact zones (Ambu et al. 2023, 2024). The previous analysis of one of these contact zones, between the subspecies *A. a. almogavarii* and *A. o. pertinax* in Catalonia, revealed geographically restricted admixture (with less than 20 km between the parental populations), which is consistent with introgressive hybridization mediated by intrinsic barriers to gene flow (Dufresnes & Martínez-Solano 2020). The partial reproductive isolation, similar lifestyle and rich bioacoustic repertoire thus offer a favorable context to document the evolution of reinforcement *in statu nascendi*.

In this study, we focus on a second hybrid zone in southern France, where the nominal subspecies *A. a. almogavarii* and *A. o. obstetricans* are expected to meet (Gonçalves et al. 2015; Ambu et al. 2023). Combining mitochondrial DNA barcoding, double digest Restriction Associated DNA sequencing (ddRAD-seq) and bioacoustic analyses, we locate the hybrid zone and test (1) whether admixture is constrained as in Catalonia, which would suggest that post-zygotic isolation between the two species also holds in this different phylogeographic setup; (2) whether the spectrum of mating calls overlaps less in the hybrid zone compared to the allopatric ranges, as expected under reinforcement; and (3) whether interspecies differences in the hybrid zone stem from character displacement.

**Methods**

*Genetic sampling*

Tissue samples were collected for 227 individuals from 76 localities (File S1), either from live animals using buccal swabs (adults; Ambu & Dufresnes 2023) or tail tips (tadpoles), or from vouchers curated in the scientific collections of BEV (CNRS – EPHE collection of the Biogeography and Ecology of the Vertebrates team in CEFE, Montpellier) and MNCN (Museo Nacional de Ciencias Naturales, Madrid). Sampling was approved by animal research ethics committees under collecting permits, namely DREAL Occitanie (N°DREAL-OCC-2023-s-05), RAVON (N°FF/75A/2016/015) and DGE-BIODIV (N°2271). Live animals were handled on site for less than a minute and tissue collection was non-destructive. Genomic DNA was isolated using an ad hoc salt protocol (Bruford et al. 1992).

*mtDNA barcoding*

We mapped the mtDNA lineages of *A. o. obstetricans* and *A. a. almogavarii* based on 619 individuals from 223 localities, combining published barcoding information (Gonçalves et al. 2015, Dufresnes & Martínez-Solano 2020, Lucati et al. 2022, Vliegenthart et al. 2022) with new sequences of the 16S rRNA gene for 221 of our samples (Files S2). To this end, a ~550 bp portion of 16S was amplified by PCR with primers 16SA-L and 16SB-H (Palumbi et al. 1991). The reaction volumes (12.5µl) involved 8.05µl of water, 2.5µl of PCR buffer, 0.25µl of dNTPs (10µM), 0.3µl of each primer (10µM), 0.1µl of Taq polymerase and 1µl of DNA template. The thermocycling program consisted of an initial denaturation of 1’30” at 94°C, 35 cycles of 45” at 94°C, 45” at 53°C, and 1’ at 72°C, followed by a final elongation of 1’ at 72°C. Amplicons were Sanger-sequenced in one direction (16SA-L). Raw sequences were quality-checked in MEGA X (Kumar et al. 2018), before manual alignment and trimming (to 530 bp) in Seaview (Gouy et al. 2021). To identify the lineages, we reconstructed a maximum-likelihood tree with PhyML 3.0 (Guindon et al. 2010).

*ddRAD-sequencing*

We analyzed ddRAD-seq data for 89 samples from 41 localities (File S1). Samples were processed in genomic libraries prepared with a custom protocol (adapted from Brelsford et al. 2016; <https://dx.doi.org/10.17504/protocols.io.kxygx3nzwg8j/v1>) and sequenced paired-end on an Illumina NextSeq 550. Raw reads were demultiplexed with STACKS 2.59 (Catchen et al. 2013) and trimmed to 2×65bp. The denovo_map.pl pipeline was applied for RAD loci construction, assembly, and cataloging with default settings. SNP calling was performed with the module *population* of STACKS to obtain the following datasets.

First, to infer the genome-average ancestry of samples, we produced a SNP matrix (–*structure*) by calling the loci shared by all 41 localities (–*p* 41) and by at least half of the samples of each locality (–*r* 0.5), while randomly keeping a single SNP per locus to avoid physically linked loci (–*write-random-snp*). The dataset contained xxx individuals genotyped at 1,642 SNPs.

Second, to measure gene flow across the hybrid zone, we produced an allele frequency dataset along a 170 km west-east geographic transect (19 localities represented by 57 samples, File S1) for loci bearing fixed species differences. To identify such loci, we first selected 20 *obstetricans* and 19 *almogavarii* reference samples, i.e., free of introgression and broadly representative of the species ranges (File S1; see Results). We exported an allele frequency dataset (–*hzar*) of 53,458 SNPs present in both reference sets (–*p* 2) and in half of the samples of each (–*r* 0.5). Of these, we flagged 10,962 SNPs with fixed different alleles between the two groups (allele frequency difference of 1). To obtain an allele frequency dataset (–*hzar*) of the flagged loci for the transect populations, we used a whitelist (–*W*) and called only the loci genotyped in all transect localities (–*p* 19) and in at least half of the samples of each locality (–*r* 0.5), retaining only one SNP per locus (–*write-random-snp*). The obtained dataset consisted of xxx individuals genotyped at 4,092 presumably unlinked species-diagnostic SNPs.

*Hybrid zone analyses*

We inferred the nuclear ancestry from the 1,642 SNPs with the clustering program STRUCTURE 2.3.4 (Pritchard et al. 2000), using *K* = 2. Several runs of 200,000 iterations after 20,000 burnin were performed to ensure convergence.

We quantified changes in allele frequency across the hybrid zone by fitting sigmoid clines on populations located along the transect using the *R* package *hzar* (Derryberry et al. 2014). We opted for models with two parameters (width *w* and center *c*) in order to compare the clines obtained for: (1) the mitogroup frequencies; (2) the STRUCTURE population ancestry *Q*; (3) the allele frequencies of 4,092 species-diagnostic SNPs.

*Bioacoustic analyses*

We recorded 406 advertisement calls from 71 individuals (2–9 calls per individual) spanning 25 localities (File S1). Recordings were made at night in June-July (the peak of breeding activity) with a PCM-A10 Sony recorder. Air temperature (T°) and the body size (snout-vent length, SVL, measured with a 1 mm precision caliper) of individuals were measured whenever possible (N = xxx individuals from yy localities), given their potential effects on temporal and spectral call parameters (Márquez & Bosch 1995).

All recordings were processed in Raven Pro 1.6.1 (The Cornell Lab, Ithaca, NY, USA). Four parameters were measured on each note and averaged by individual: (1) the dominant frequency DF (Hz), as the frequency of the greatest energy in the note; (2) the note duration ND (s), as the time between the first and last pulses of the note, (3) the rising time RT (s), as the time between the first pulse and the pulse with the highest frequency; (4) the pulse rate PR (s^-1^), as the number of pulses per second. See File S3 for a graphical display.

SVL had no effect on any bioacoustic variable (Pearson’s product-moment correlations, *P* = 0.35–0.94) but T° significantly influenced ND (*P* < 0.005, *r* = -0.64). To alleviate this effect, we used the residuals of the linear regression T°~ND in downstream analyses. Individuals were assigned to four groups according to their species and geographic origin: allopatric *obstetricans* (*n* = 27), allopatric *almogavarii* (*n* = 22); parapatric *obstetricans* (*n* = 9); parapatric *almogavarii* (*n* = 13). Variation among groups was assessed by a Multivariate Analysis of Variance (MANOVA) and a Principal Component Analysis (PCA), building convex hulls to visualize group variation and overlap.

We performed three ad hoc statistical tests to explore specific expectations on the patterns of variation between species and allopatric/parapatric individuals under reinforcement and its underlying signatures (Fig. 1). First, we compared hull overlaps between species, which under reinforcement should be lower for parapatric than allopatric individuals. To this end, we used computed hull overlap with the *Overlap* function of the R package *shipunov* (Shipunov, 2020). This was done separately for the two pairs of PCs (PC1/PC2 and PC3/PC4), and averaged over PCs, weighing for their relative contributions. Second, we compared hulls’ centroid distances between species, which under character displacement should be higher between the parapatric than the allopatric individuals (left part of Fig. 1). To this end, we measured the centroid (mean coordinate) of each group for each PC, weighted them by their relative contribution, and computed their pairwise Euclidian distance with the *dist* function of the R package *stats*. Third, we compared call diversity between the two species, which is expected to be lower among parapatric than allopatric individuals if between-species overlap is minimized by a reduction of the call repertoire (right part of Fig. 1). To this end, we calculated the standard deviation of each group for each PC and weighed them by their relative contribution.

For each test, we computed relevant statistics, namely (1) the difference ∆ between the allopatric and parapatric *almogavarii*-*obstetricans* overlaps; (2) the difference ∆ between the allopatric and parapatric *almogavarii*-*obstetricans* centroid distances; (3) the differences ∆ between the standard deviations of the parapatric and allopatric populations of each species, as well as the cumulated differences over both species. To assess statistical significance, we designed a permutation test that reshuffles group assignments 1,000 times. Because the sample sizes differed between groups, we performed a second set of permutations in which the two largest groups were randomly down-sampled to 10 individuals. All statistical analyses were conducted in R.

**Results and Discussion**

Mitochondrial barcoding and nuclear clustering analyses successfully distinguish *A. obstetricans* and *A. almogavarii* and provide concordant patterns regarding their respective distributions in southern France (Fig. 2A–B; File S4). We located at least two transitions: one in the Montagne Noire area (between locs 32 and 33) and one near the Ariège river in the Pyrenean foothills (between locs 10 and 11). The latter, which we densely sampled along a geographic transect, features traces of genetic admixture (and thus introgressive hybridization) on both sides of the river (locs 6–15). The mitochondrial and nuclear genome average clines had steep average widths *w* of 9.8 km (95% confidence interval: 6.4–17.1) and 20.6 km (12.5–41.4), with broadly matching centers *c* (~5 km apart) (Fig. 2C, File S5). Clines inferred for individual SNPs broadly follow the genome average (*w* = 21.2 ± 9.5 km; Fig. 2C–D) with relatively homogeneous variation in both *w* (~5–40km) and *c* (+/- ~10km from the average *c*), except for a few SNPs where *w* is essentially null (Fig. 2D).

These results largely corroborate observations from the Catalonian hybrid zone, where the *A. obstetricans*/*almogavarii* transition is also characterized by steep clines (16 km on average) and a few loci entirely resist introgression (Dufresnes & Martínez-Solano 2020). These likely represent barrier loci, i.e., potentially linked to genes involved in Dobzhansky-Muller hybrid incompatibilities (as documented in other anuran hybrid zones; Dufresnes et al. 2021). Replicate hybrid zones between the same species pairs sometimes show contrasting outcomes, depending on intrinsic and extrinsic factors (e.g., Dufresnes et al. 2020, Velo-Antón et al. 2021). Instead, here *A. obstetricans* and *A. almogavarii* barely admix in both surveyed areas, regardless of the *A. obstetricans* lineages involved (subspecies *pertinax* in Catalonia vs. subspecies *obstetricans* in France), thus confirming their species status (Speybroeck et al. 2020).

In parallel, we found significant differences in the mating calls of the two species (MANOVA, *F* = 4.1, dof = 66, *P* = 0.005). Most of these differences are driven by PC1 in the PCA (Fig. 3), which reflects the pulse rate (PR) and the dominant frequency (DF) – both being generally higher in *A. almogavarii* (File S6). The two species’ calls significantly overlap less in the parapatric than the allopatric populations (Table 1, Fig. 3). However, this reduced overlap is not due to a shift in the average call parameters: the interspecific call differences are not significantly greater between the parapatric and allopatric populations (Table 1). Instead, the parapatric individuals feature lesser call variation than the allopatric individuals, a trend that is marginally significant for *A. almogavarii*, and when combining both species together (Table 1). The same patterns are also obtained when the most represented groups are down-sampled in the permutation analyses (Table 1).

The genomic and bioacoustic variation observed thus comply with two of the general expectations of reinforcement: (1) that the hybrid zone is putatively maintained by partial reproductive isolation; and (2) that a reproductive cue (here, the advertisement calls) is more dissimilar between the two species in the populations from inside than outside of the hybrid zone. In addition, the dissimilarities reported here reflect a reduction of the species’ standing variation (Fig. 1, right) rather than a disruptive character displacement (Fig. 1, left). Reinforcement is a form of local adaptation (to the presence of “wrong” mates). Therefore, assuming that mating call patterns in our midwife toad hybrid zone are driven by reinforcement, our observations are reminiscent of local adaptation literature, which accordingly assigns an important role to the fixation of existing variants rather than the evolution of new ones (e.g., Cayuela et al., 2020).

That being said, it is too preliminary to affirm that *A. obstetricans* and *A. almogavarii* are experiencing reinforcement, given that the documented bioacoustic variation may have also been influenced by other processes. Anuran calls are known to depend on various biotic and abiotic factors (Köhler et al. 2017), related to e.g., meteorology (Oseen et al. 2022), microhabitat (Ziegler et al. 2011), geography (Weaver et al. 2020), predation pressure (Akre et al. 2011) and even anthropological disturbance (Liu et al. 2022). Due to their geographic proximity, our parapatric populations inevitably share more similar environmental conditions than the more scattered allopatric populations, which could have led to the observed patterns of call variability. These patterns may also be a neutral consequence of introgressive hybridization: admixture in the parapatric populations homogenizes heritable phenotypes (thus preventing character displacement) while reducing individual fitness due to hybrid incompatibilities (thus decreasing effective population size and phenotypic variability). Future work should aim at quantifying assortative mating and the potential fitness costs of hybridization directly, as well as establishing causality links with the observed variation at mating calls, for instance, under experimental setups. Moreover, reinforcement is expected to leave peaks of genomic differentiation between sympatric and allopatric individuals that are shared across both species, notably around the genes involved in mate-recognition (Garner et al. 2018). Such signature may be screened from our ddRAD-seq data, providing that the loci can be mapped to long-read assemblies such as a reference genome.

To date, reinforcement has been primarily examined in reproductively well-isolated species that form bimodal hybrid zones i.e., where hybridization and introgression is drastically restricted, and distinct hybrid/parental categories co-exist. Some of these cases have been criticized because character displacement between deeply-diverged species can be explained by alternative processes (e.g., ecological adaptations that evolved before the contact, Noor, 1999; Coyne & Orr, 2004; runaway sexual selection, Day, 2000). Our study highlights that younger, lesser differentiated species that form unimodal hybrid zones, i.e., where large parts of the genomes remain permeable to gene flow, can in principle feature the genetic and phenotypic variation necessary to evolve reinforcement, even without obvious character displacement. The empirical focus on such systems should thus widen our understanding of the phenomenon across broader evolutionary contexts.

**Author’s contributions**: JA conducted fieldwork, labwork, analyses and wrote the first draft of the paper; CD conducted fieldwork, supervised the study, and edited the paper.

**Acknowledgements –** We are grateful to O. Buisson, P-A. Crochet, C. Delmas, M. Denoël, N. Fuento, P. Geniez, M. Gilbert, O. Hadj-Bachir, P. Lemmers, I. Martínez-Solano, T. Suchan, A. Trochet, M. Vences and B. Wielstra for discussion, lab support and sample collection/access.

**Funding** – This study was funded by the Taxon-Omics priority program (SPP1991) of the Deutsche Forschungsgemeinschaft (grant N° VE247/19-1 to CD) and by the National Natural Science Foundation of China (RFIS grant N° 3211101356 to CD).

**Conflict of interest** – The authors declare no conflicts of interest.

**Ethics statement** – Sampling was approved by animal research ethics committees under collecting permits, namely DREAL Occitanie (N°DREAL-OCC-2023-s-05), RAVON (N°FF/75A/2016/015) and DGE-BIODIV (N°2271). Live animals were handled on site for less than a minute and tissue collection was non-destructive.

**Data availability statement** – 16S sequences are publicly available on GenBank (accessions OR725791–OR725973 and OR916149–OR916186); raw ddRAD sequence reads are archived on NCBI SRA under BioProject PRJNA949685; analyzed datasets (16S alignment, SNP data, bioacoustic data) are available on Zenodo (doi: 10.5281/zenodo.10037218).

**References**

Akre KL, Farris HE, Lea AM, Page RA, Ryan MJ. 2011. Signal perception in frogs and bats and the evolution of mating signals. *Science* 333: 751–752.

Ambu J, Dufresnes C. 2023. Buccal swabs for amphibian genomics. *Amphibia-Reptilia* 44: 249–255.

Ambu J, Martínez-Solano Í, Suchan T, Hernandez A, Wielstra B, Crochet P-A, Dufresnes C. 2023. Genomic phylogeography illuminates deep cyto-nuclear discordances in midwife toads (*Alytes*). *Molecular Phylogenetics and Evolution* 183: 107783.

Baiz MD, Tucker PK, Cortés-Ortiz L. 2019. Multiple forms of selection shape reproductive isolation in a primate hybrid zone. *Molecular Ecology* 28: 1056–1069.

Blair WF. 1964. Isolating mechanisms and interspecies interactions in anuran amphibians. *The Quarterly Review of Biology* 39: 334–344.

Brelsford A, Dufresnes C, Perrin N. 2016. High-density sex-specific linkage maps of a European tree frog (*Hyla arborea*) identify the sex chromosome without information on offspring sex. *Heredity* 116: 177–181.

Bruford MW, Hanotte O, Brookfield JFY, Burke T. 1992. Single-locus and multilocus DNA fingerprinting. 225–269. In: HOELZEL, A. R. 1 Ed. Molecular genetic analyses of populations: A Practical Approach. IRL Press, Oxford. 315 pp.

Butlin R. 1987. Speciation by reinforcement. *Trends in Ecology & Evolution* 2: 8–13.

Butlin R, Smadja CM. 2018. Coupling, reinforcement, and speciation. The American Naturalist 191: 155–172.

Catchen J, Hohenlohe PA, Bassham S, Amores A, Cresko WA. 2013. Stacks: an analysis tool set for population genomics. *Molecular Ecology* 22: 3124–3140.

Cayuela H, Rougemont Q, Laporte M, Mérot C, Normandeau E, Dorant Y, Tørresen OK, Khang Hoff SN, Jentoft S, Sirois P, Castonguay M, Jansen T, Praebel K, Clément M, Bernatchez L. 2020. Shared ancestral polymorphisms and chromosomal rearrangements as potential drivers of local adaptation in a marine fish. *Molecular Ecology* 29: 2379–2398.

Coyne JA, Orr HA. 1989. Patterns of speciation in *Drosophila*. *Evolution* 43: 362–381.

Coyne JA, Orr HA. Speciation. 2004. Sinauer Associates, Sunderland, Massachusetts. 545 pp.

Day T. 2000. Sexual selection and the evolution of costly female preferences: Spatial effects. *Evolution* 54: 715–730.

Derryberry EP, Derryberry GE, Maley JM, Brumfield RT. 2014. HZAR: hybrid zone analysis using an R software package. *Molecular Ecology Resources* 14: 652–663.

de Solan T, Théry M, Picard D, Crochet PA, David P, Secondi J. 2022. A lot of convergence, a bit of divergence: environment and interspecific interactions shape body color patterns in *Lissotriton* newts. *Journal of Evolutionary Biology* 35: 575–588.

Dobzhansky T. 1937. Genetics and the origin of species. Columbia University Press, New York. 364 pp.

Dobzhansky T. 1940. Speciation as a stage in evolutionary divergence. *The American Naturalist* 74: 312–321.

Dufresnes C, Martínez-Solano Í. 2020. Hybrid zone genomics supports candidate species in Iberian *Alytes obstetricans*. *Amphibia-Reptilia* 41: 105–112.

Dufresnes C, Berroneau M, Dubey S, Litvinchuk SN, Perrin N. 2020. The effect of phylogeographic history on species boundaries: a comparative framework in *Hyla* tree frogs. *Scientific Reports*, 10: 5502.

Dufresnes C, Brelsford A, Jeffries DL, Mazepa G, Suchan T, Canestrelli D, Nicieza A, Fumagalli L, Dubey S, Martínez‐Solano Í, Litvinchuk S, Vences M, Perrin N, Crochet P-A. 2021. Mass of genes rather than master genes underlie the genomic architecture of amphibian speciation. *Proceedings of the National Academy of Sciences* 118: e2103963118.

Fouquette M .1960. Isolating mechanisms in three sympatric treefrogs in the canal zone. *Evolution* 14: 484–97.

Garner A, Goulet B, Farnitano M, Molina-Henao Y, Hopkins R. 2018. Genomic signatures of reinforcement. *Genes* 9: 191.

Gonçalves H, Maia-Carvalho B, Sousa-Neves T, García-París M, Sequeira F, Ferrand N, Martínez-Solano Í. (2015). Multilocus phylogeography of the common midwife toad, *Alytes obstetricans* (Anura, Alytidae): contrasting patterns of lineage diversification and genetic structure in the Iberian refugium. *Molecular Phylogenetics and Evolution* 93: 363–379.

Gouy M, Tannier E, Comte N, Parsons DP. 2021. Seaview version 5: a multiplatform software for multiple sequence alignment, molecular phylogenetic analyses, and tree reconciliation. *Methods in Molecular Biology* 2231: 241–2610.

Guindon S, Dufayard JF, Lefort V, Anisimova M, Hordijk W, Gascuel O. 2010. PhyML 3.0: new algorithms, methods and utilities. *Systematic Biology* 59: 307–321.

Hewitt GM. 1988. Hybrid zones-natural laboratories for evolutionary studies. *Trends in Ecology & Evolution* 3: 158–167.

Higgie M, Chenoweth S, Blows MW. 2000. Natural selection and the reinforcement of mate recognition. *Science* 290: 519–521.

Hoffmann A, Reyer H-U. 2013. Genomic effects on advertisement call structure in diploid and triploid hybrid waterfrogs (Anura, *Pelophylax esculentus*). *BMC Ecology* 13: 47.

Hopkins R. 2013. Reinforcement in plants. *New Phytologist* 197: 1095–1103.

Hoskin CJ, Higgie M, McDonald KR, Moritz C. 2005. Reinforcement drives rapid allopatric speciation. *Nature* 437: 1353–1356.

Hostert EE. 1997. Reinforcement: A new perspective on an old controversy. *Evolution* 51: 697–702.

Howard DJ. 1993. Reinforcement: origin, dynamics, and fate of an evolutionary hypothesis. In Hybrid zones and the evolutionary process. Edited by R. G. Harrison, 46–69. Oxford University Press, New York.

Kay KM, Schemske DW. 2008. Natural selection reinforces speciation in a radiation of neotropical rainforest plants. *Evolution* 62: 2628–2642.

Kime NM, Turner WR, Ryan MJ. 2000. The transmission of advertisement calls in Central American frogs. *Behavioral Ecology* 11: 71–83.

Köhler J, Jansen M, Rodríguez A, Kok PJ, Toledo LF, Emmrich M, Glaw F, Haddad CFB, Rödel M-O, Vences M. (2017). The use of bioacoustics in anuran taxonomy: theory, terminology, methods and recommendations for best practice. *Zootaxa* 4251: 1–124.

Kronforst MR, Young LG, Gilbert LE. 2007. Reinforcement of mate preferences among hybridizing *Heliconius* butterflies. *Journal of Evolutionary Biology* 20: 278–285.

Kumar S, Stecher G, Li M, Knyaz C, Tamura K. 2018. MEGA X: molecular evolutionary genetics analysis across computing platforms. *Molecular Biology & Evolution* 35: 1547–1549.

Lange L, Brischoux F, Lourdais O. 2022. Benefits of paternal thermoregulation: male midwife toads select warmer temperature to shorten embryonic development. *Behavioral Ecology and Sociobiology* 76: 48.

Lemmon AR, Smadja C, Kirkpatrick M. 2004. Reproductive character displacement is not the only possible outcome of reinforcement. *Journal of Evolutionary Biology* 17: 177–183.

Lemmon EM. 2009. Diversification of conspecific signals in sympatry: geographic overlap drives multidimensional reproductive character displacement in frogs. *Evolution* 63: 1155–1170.

Lemmon EM, Juenger TE. 2017. Geographic variation in hybridization across a reinforcement contact zone of chorus frogs (*Pseudacris*). *Ecology and Evolution* 7: 9485–9502.

Liu N, Zhong S, Wang T, Li X, Wei L, Zou C, Zhao S, Li B. 2022. Advertisement call variation of two frog species along an urban-rural gradient in Shanghai, China. *Diversity* 14: 550.

Lucati F, Miró A, Bosch J, Caner J, Jowers MJ, Rivera X, Donaire-Barroso D, Rebelo R, Ventura M. 2022. New insights on patterns of genetic admixture and phylogeographic history in Iberian high mountain populations of midwife toads. *PLOS one* 17: e0277298.

Lukhtanov VA. 2011. Dobzhansky’s rule and reinforcement of prezygotic reproductive isolation in zones of secondary contact. *Biology Bulletin Reviews* 1: 2–12.

Márquez R. Bosch J. 1995. Advertisement calls of the midwife toads *Alytes* (Amphibia, Anura, Discoglossidae) in continental Spain. *Journal of Zoological Systematics and Evolutionary Research* 33: 185–192.

Noor MAF. 1995. Speciation driven by natural selection in *Drosophila*. *Nature* 375: 674–675.

Noor MAF. 1999. Reinforcement and other consequences of sympatry. *Heredity* 83: 503–508.

Nosil P, Crespi BJ, Sandoval CP. 2003. Reproductive isolation driven by the combined effects of ecological adaptation and reinforcement. *Proceedings of the Royal Society B: Biological Sciences* 270: 1911–1918.

Ortiz-Barrientos D, Counterman BA, Noor MAF. 2004. The genetics of speciation by reinforcement. *PLOS Biology* 2: 2256–2263.

Oseen KL, Wassersug RJ. 2002. Environmental factors influencing calling in sympatric anurans. *Oecologia* 133: 616–625.

Palumbi SR, Martin A, Romano S, McMillan WO, Stice L, Grabowski G. 1991. The Simple Fool's Guide to PCR, Version 2.0. Privately published, University of Hawaii, Manoa. 47 pp.

Pellegrino G. 2016. Sympatric reinforcement of reproductive barriers between *Neotinea tridentata* and *N. ustulata* (Orchidaceae). *Journal of Plant Research* 129: 1061–1068.

Peterson MA, Honchak BM, Locke SE, Beeman TE, Mendoza J, Green J, Buckingham KJ, White MA, Monsen KJ. 2005. Relative abundance and the species-specific reinforcement of male mating preference in the *Chrysochus* (Coleoptera: Chrysomelidae) hybrid zone. *Evolution* 59: 2639–2655.

Pfennig KS, Simovich MA. 2002. Differential selection to avoid hybridization in two toad species. *Evolution* 56: 1840–1848.

Pfennig KS. 2003. A test of alternative hypotheses for the evolution of reproductive isolation between spadefoot toads: support for the reinforcement hypothesis. *Evolution* 57: 2842–2851.

Pfennig KS, Rice AM. 2014. Reinforcement generates reproductive isolation between neighbouring conspecific populations of spadefoot toads. *Proceedings of the Royal Society B: Biological Sciences* 281: 20140949.

Porretta D, Urbanelli S. 2012. Evolution of premating reproductive isolation among conspecific populations of the sea rock-pool beetle *Ochthebius urbanelliae* driven by reinforcing natural selection. *Evolution* 66: 1284–1295.

Pritchard JK, Stephens M, Donnelly P. 2000. Inference of population structure using multilocus genotype data. *Genetics* 155: 945–959.

Ramsey J,Bradshaw HD, Schemske DW. 2003. Components of reproductive isolation between the monkeyflowers *Mimulus lewisii* and *M. cardinalis* (Phrymaceae). *Evolution* 57: 1520–1534.

Roda F, Mendes FK, Hahn MW, Hopkins R. 2017. Genomic evidence of gene flow during reinforcement in Texas Phlox. *Molecular Ecology* 26: 2317–2330.

Rundle HD, Schluter D. 1998. Reinforcement of stickleback mate preferences: sympatry breeds contempt. *Evolution* 52: 200–208.

Saetre G, Moum T, Bures S, Kral, Adamjan M, Moreno J. 1997. A sexually selected character displacement in flycatchers reinforces premating isolation. *Nature* 387: 589–592.

Sebastianelli M, Lukhele S, Secomandi S, de Souza S, Haase B, Moysi M, Nikiforou C, Hutfluss A, Mountcastle J, Balacco J, Pelan S, Chow W, Fedrigo O, Downs C, Monadjem A, Dingemanse N, Jarvis E, Brelsford A, vonHorldt B, Krirschel A. 2023. A role for vocal rhythm in avian speciation. PREPRINT (Version 1) available at Research Square [https://doi.org/10.21203/rs.3.rs-3175064/v1]

Servedio MR, Noor MAF. 2003. The role of reinforcement in speciation. Theory and data. *Annual Review of Ecology, Evolution, & Systematics* 34: 339–364.

Shipunov A. 2020. Visual statistics. Use R! July 27, 2020 version. 451 pp.

Silvertown J, Servaes C, Biss P, Macleod D. 2005. Reinforcement of reproductive isolation between adjacent populations in the Park Grass Experiment. *Heredity* 95: 198–205.

Smadja C, Ganem G. 2005. Asymmetrical reproductive character displacement in the house mouse. *Journal of Evolutionary Biology* 18: 1485–1493.

Souza NA, Andrade-Coelho CA, Vigoder FM, Ward RD, Peixoto AA. 2008. Reproductive isolation between sympatric and allopatric Brazilian populations of *Lutzomyia longipalpis* sl (Diptera: Psychodidae). *Memórias do Instituto Oswaldo Cruz* 103: 216–219.

Speybroeck J, Beukema W, Dufresnes C, Fritz U, Jablonski D, Lymberakis P, Martínez‐Solano Í, Razzetti E, Vamberger M, Vences M, Vörös J, Crochet P-A. 2020. Species list of the European herpetofauna–2020 update by the Taxonomic Committee of the Societas Europaea Herpetologica. *Amphibia-Reptilia* 41: 139–189.

St. John ME, Fuller RC. 2021. Asymmetric reinforcement in Lucania killifish: assessing reproductive isolation when both sexes choose. *Current Zoology* 67: 215–224.

Twomey E, Vestergaard JS, Summers K. 2014. Reproductive isolation related to mimetic divergence in the poison frog *Ranitomeya imitator*. *Nature Communications* 5: 4749.

Vallin N, Rice AM, Bailey RI, Husby A, Qvarnström A. 2012. Positive feedback between ecological and reproductive character displacement in a young avian hybrid zone. *Evolution* 66: 1167–1179.

Velo-Antón G, Lourenço A, Galán P, Nicieza A, Tarroso P. 2021. Landscape resistance constrains hybridization across contact zones in a reproductively and morphologically polymorphic salamander. *Scientific Reports* 11: 9259.

Vliegenthart C, van de Vrede M, den Boer I, Gilbert MJ, Lemmers P, France J, de Visser MC, Struijk RPJH, & Wielstra B. 2022. The limits of mtDNA analysis for determining the provenance of invasive species: a midwife toad example. *Amphibia-Reptilia* 44: 27–33.

Weaver SJ, Callaghan CT, Rowley JJ. 2020. Anuran accents: Continental‐scale citizen science data reveal spatial and temporal patterns of call variability. *Ecology and Evolution* 10: 12115–12128.

Yukilevich, R. 2012. Asymmetrical patterns of speciation uniquely support reinforcement in *Drosophila*. *Evolution* 66:1430–1446.

Yukilevich R. 2021. Reproductive character displacement drives diversification of male courtship songs in *Drosophila*. *The American Naturalist* 197: 690–707.

Ziegler L, Arim M, Narins PM. 2011. Linking amphibian call structure to the environment: the interplay between phenotypic flexibility and individual attributes. *Behavioral Ecology* 22: 520–526.

**Tables**

**Table 1:** Comparison of the bioacoustic variation between allopatric and parapatric species across the four axes of the PCA (Fig. 3). *P* indicates the proportion of permutations consistent with the observed pattern (brackets: permutations subsetting the largest groups).

|  | **allopatric** | **parapatric** | **∆** | ***P*** |
| --- | --- | --- | --- | --- |
|  |  |  |  |  |
| Overlap between species | 0.82 | 0.33 | -0.49 | **0.022** (**0.005**) |
| Centroid distance between species | 1.47 | 1.79 | +0.32 | 0.456 (0.269) |
| Standard deviation – *obstetricans* | 3.53 | 2.97 | -0.57 | 0.235 (0.271) |
| Standard deviation – *almogavarii* | 3.68 | 2.54 | -1.14 | **0.017** (**0.047**) |
| Standard deviation – cumulated | 7.21 | 5.51 | -1.71 | **0.029** (**0.048**) |
|  |  |  |  |  |
|  |  |  |  |  |

**Figures**

**Fig. 1: Two hypotheses for the divergence of reproductive traits expected under reinforcement.** While allopatric populations of two species (plain ellipses and +) may retain widely overlapping variation for reproductive traits, reinforcement predicts a reduction of this overlap in the hybrid zone (empty ellipses and ×) as a selective response against maladaptive hybridization. This can be achieved by reproductive character displacement, which increases mean interspecific differences (left), or by reducing intraspecific variation without shifting the average phenotypes (right).

**Fig. 2: Genetic structure of the *A. o. obstetricans* / *A. a. almogavarii* hybrid zone.** (A) Distributions of the mitochondrial lineages. (B) Individual (barplots) and population (piecharts on the map) nuclear ancestry estimated from 1,642 unlinked SNPs. (C) Sigmoid clines fitted on mitogroup frequencies (dashed cline), average ancestries (black plain cline, with the 95% confidence interval) and frequencies of 4,092 species-diagnostic SNPs (grey clines) along the geographic transect. (D) Variation in cline center (as the deviation ∆*c* from the average center) and cline width *w*. Arrows point to potential barrier loci (null width).

**Fig. 3: Bioacoustic variation among 71 individuals in (parapatric) and out (allopatric) of the hybrid zone.** (A) PCA on four mating call parameters. (B) Geographic origin and group assignments of calls.

**Supplementary material captions –** File S1: list of samples used in this study; File S2: list of localities barcoded with mtDNA; File S3: bioacoustic scheme; File S4: 16S tree; File S5: cline parameters; File S6: summary statistics of the call parameters.
