# Supplementary material for: Genomic and bioacoustic variation in a midwife toad hybrid zone: A role for reinforcement?
Source: PLoS One. 2024 Nov 25;19(11):e0314477. doi: 10.1371/journal.pone.0314477 (PMC11588267; doi:10.1371/journal.pone.0314477)
Supplement: Attachment — Submitted filename: Responses_to_Reviewers.R1.docx [file pone.0314477.s008.docx]

**Response to Referees**

Copyright queries (sent 11 october 2024 by Richard Ibañez Dilla)

1. Thank you for confirming that the map images in Figures 2 and 3 were created with shapefiles from Natural Earth and are in the public domain. However, please note that we require additional information about the other image in Figure 2 (i.e. frog image). Before we proceed, please respond to the remaining copyright queries:

1) Please explain where the authors obtained the image of a frog in Figure 2 in your submission. If the authors created this image themselves, please confirm this in your response.

R: The image was created by us. We have now specified this in the legend of Figure 2 by adding the photo credit (J. Ambu, the first author).

2) Please state whether the image of a frog in Figure 2 has been previously published or copyrighted. Note: If the authors created this image themselves and this image has not been previously copyrighted, please disregard question (3) below.

R: We confirm that the image has not been previously published or copyrighted.

Journal Requirement queries:

When submitting your revision, we need you to address these additional requirements.

1. Please ensure that your manuscript meets PLOS ONE's style requirements, including those for file naming. The PLOS ONE style templates can be found at 
https://journals.plos.org/plosone/s/file?id=wjVg/PLOSOne_formatting_sample_main_body.pdf and 
https://journals.plos.org/plosone/s/file?id=ba62/PLOSOne_formatting_sample_title_authors_affiliations.pdf

R: We have accordingly revised our entire manuscript to match the journal’s style following the tutorial files above.

2. Please clarify the issuing authority of the permits obtained, including spelling out any acronyms. Please also specified the fate of all animals used - whether released back in the environment or something else, and whether any animals died during the work.

R: We have spelled out the issuing authorities of the permits and clarified that animals were released to their place of capture immediately after handling in the Material and Methods section.

3. Thank you for stating the following financial disclosure: 
"This study was funded by the Taxon-Omics priority program (SPP1991) of the Deutsche Forschungsgemeinschaft (grant N° VE247/19-1 to CD) and by the National Natural Science Foundation of China (RFIS grant N° 3211101356 to CD)."

Please state what role the funders took in the study.  If the funders had no role, please state: "The funders had no role in study design, data collection and analysis, decision to publish, or preparation of the manuscript." 
If this statement is not correct you must amend it as needed. 
Please include this amended Role of Funder statement in your cover letter; we will change the online submission form on your behalf.

R: The funders took no part in the study and we have added the above statement in our cover letter, as suggested.

4. Your ethics statement should only appear in the Methods section of your manuscript. If your ethics statement is written in any section besides the Methods, please delete it from any other section.

R: We have now removed any other mention of the ethics statement apart from the Material and methods section, as requested.

5. We note that [Figures 2 and 3] in your submission contain [map/satellite] images which may be copyrighted. All PLOS content is published under the Creative Commons Attribution License (CC BY 4.0), which means that the manuscript, images, and Supporting Information files will be freely available online, and any third party is permitted to access, download, copy, distribute, and use these materials in any way, even commercially, with proper attribution. For these reasons, we cannot publish previously copyrighted maps or satellite images created using proprietary data, such as Google software (Google Maps, Street View, and Earth). For more information, see our copyright guidelines: http://journals.plos.org/plosone/s/licenses-and-copyright.
 
We require you to either (1) present written permission from the copyright holder to publish these figures specifically under the CC BY 4.0 license, or (2) remove the figures from your submission:
 
a. You may seek permission from the original copyright holder of Figures 2 and 3 to publish the content specifically under the CC BY 4.0 license.  
 
We recommend that you contact the original copyright holder with the Content Permission Form (http://journals.plos.org/plosone/s/file?id=7c09/content-permission-form.pdf) and the following text:
“I request permission for the open-access journal PLOS ONE to publish XXX under the Creative Commons Attribution License (CCAL) CC BY 4.0 (http://creativecommons.org/licenses/by/4.0/). Please be aware that this license allows unrestricted use and distribution, even commercially, by third parties. Please reply and provide explicit written permission to publish XXX under a CC BY license and complete the attached form.”
 
Please upload the completed Content Permission Form or other proof of granted permissions as an "Other" file with your submission.
 
In the figure caption of the copyrighted figure, please include the following text: “Reprinted from [ref] under a CC BY license, with permission from [name of publisher], original copyright [original copyright year].”
 
b. If you are unable to obtain permission from the original copyright holder to publish these figures under the CC BY 4.0 license or if the copyright holder’s requirements are incompatible with the CC BY 4.0 license, please either i) remove the figure or ii) supply a replacement figure that complies with the CC BY 4.0 license. Please check copyright information on all replacement figures and update the figure caption with source information. If applicable, please specify in the figure caption text when a figure is similar but not identical to the original image and is therefore for illustrative purposes only.
The following resources for replacing copyrighted map figures may be helpful:
 
USGS National Map Viewer (public domain): http://viewer.nationalmap.gov/viewer/
The Gateway to Astronaut Photography of Earth (public domain): http://eol.jsc.nasa.gov/sseop/clickmap/
Maps at the CIA (public domain): https://www.cia.gov/library/publications/the-world-factbook/index.html and https://www.cia.gov/library/publications/cia-maps-publications/index.html
NASA Earth Observatory (public domain): http://earthobservatory.nasa.gov/
Landsat: http://landsat.visibleearth.nasa.gov/
USGS EROS (Earth Resources Observatory and Science (EROS) Center) (public domain): http://eros.usgs.gov/#
Natural Earth (public domain): <http://www.naturalearthdata.com/>

R: These maps were built by us based on background shapefile layers obtained from Natural Earth (public domain) so this is not an issue. In its terms of use, the Natural Earth website accordingly states “No permission is needed to use Natural Earth. Crediting the authors is unnecessary.”. We therefore did not change these figures nor their legend in our revision.

6. Please include captions for your Supporting Information files at the end of your manuscript, and update any in-text citations to match accordingly. Please see our Supporting Information guidelines for more information: http://journals.plos.org/plosone/s/supporting-information.

R: We have provided captions for SI files following the guidelines and double-checked that these match in-text citation, accordingly.

7. Please review your reference list to ensure that it is complete and correct. If you have cited papers that have been retracted, please include the rationale for doing so in the manuscript text, or remove these references and replace them with relevant current references. Any changes to the reference list should be mentioned in the rebuttal letter that accompanies your revised manuscript. If you need to cite a retracted article, indicate the article’s retracted status in the References list and also include a citation and full reference for the retraction notice.

R: We have reviewed our reference list accordingly and did not add or remove any citation.

Additional Editor Comments:

Having considered the feedback from both reviewers, I am in agreement that the manuscript can be accepted after minor revisions.

R: Thank you. We have conducted the requested minor revisions.

Review Comments to the Author

Reviewer #1: The study focuses on patterns of genomic and bioacoustic differentiation in a hybrid zone involving two species of midwife toads. The data are comprehensive and well suited to tests hypotheses about the role of reinforcement in the maintenance of species boundaries. The analyses are state of the art and the results are discussed appropriately, including potential shortcomings and future directions. I have made my suggestions, all minor, directly on the Word file (attached).

R: Thank you for your positive feedback and in-text comments. We have considered all of them in the revisions, with our specific answers provided in your file.

Reviewer #2: The paper is very interesting and well written. Hybrid zones provide good opportunities to understand mechanisms of reproductive isolation and speciation. The paper examines one of the important aspects - the reinforcement (by which reproductive barriers should be increase). Using mitochondrial barcoding, population genomics, and bioacoustic analysis, the authors studied the hybrid zone between two closely related amphibian species. It turned out that the breeding calls of the species overlap less inside than outside the hybrid zone. Perhaps, this is due to a reduction of their standing variation rather than a shift towards distinctive variants. This finding is very important for assessing the genetic and behavioral factors that contribute to reproductive isolation.
I did not find any inconsistencies. Therefore, I recommend it for publication in this journal.

R: Thank you for your positive feedback and recommendation of acceptance without revisions.
