# Supplementary figures and images for: Correction: Genomic and bioacoustic variation in a midwife toad hybrid zone: A role for reinforcement?
Source: PLoS One. 2025 Dec 1;20(12):e0337571. doi: 10.1371/journal.pone.0337571 (PMC12668509; doi:10.1371/journal.pone.0337571)

**S1 Fig. Oscillogram (top) and spectrogram (bottom) of a note, showing the variables measured.**


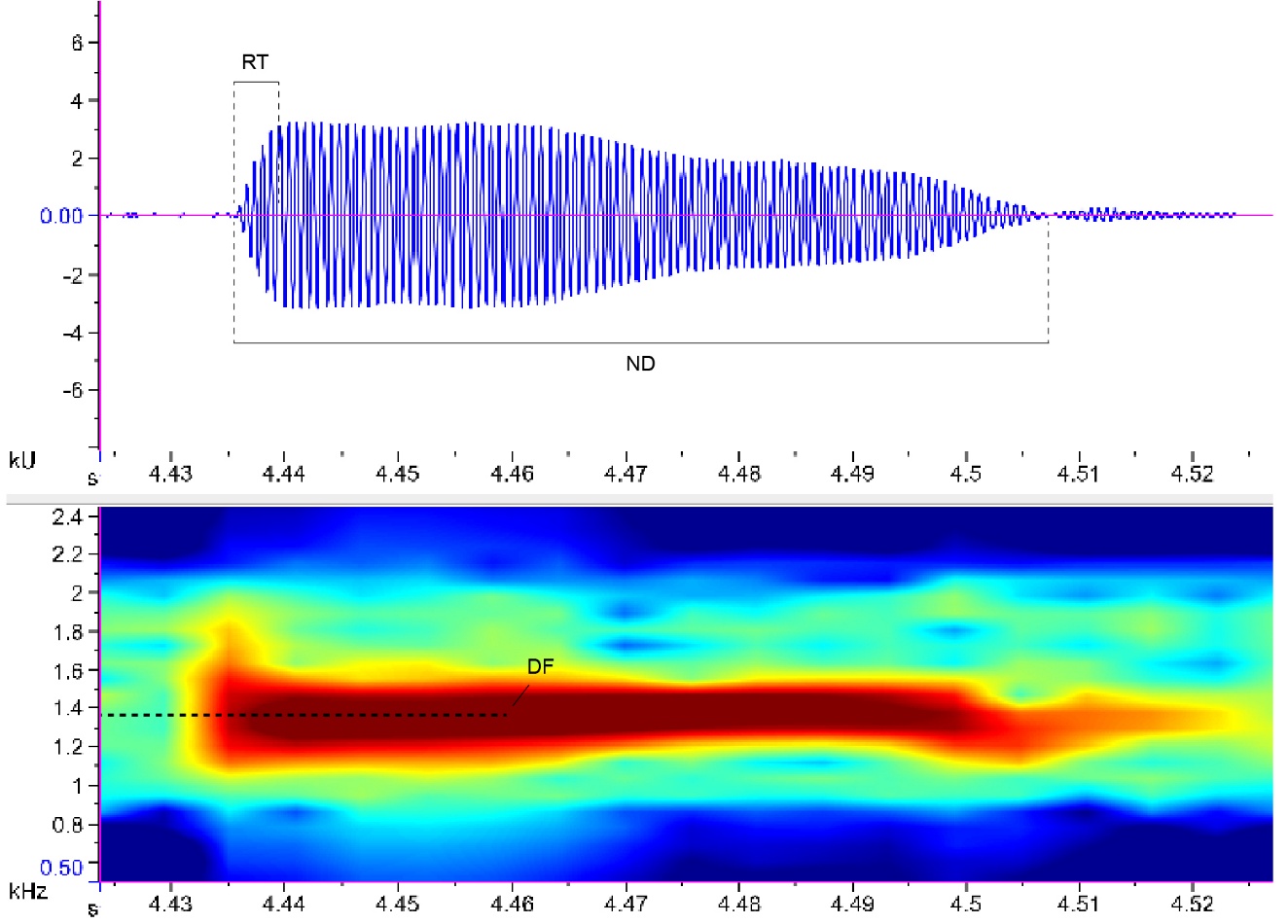

Supplement: S1 Fig — (DOCX) [file pone.0337571.s001.docx]
