# Supplementary material for: Correction: Genomic and bioacoustic variation in a midwife toad hybrid zone: A role for reinforcement?
Source: PLoS One. 2025 Dec 1;20(12):e0337571. doi: 10.1371/journal.pone.0337571 (PMC12668509; doi:10.1371/journal.pone.0337571)
Supplement: S4 Table — DF: dominant frequency; ND: note duration; RT: rising time. (DOCX) [file pone.0337571.s002.docx]

**S4 Table. Mean, standard variation (±) and range (in brackets) of the bioacoustic variables in the four groups.** DF: dominant frequency; ND: note duration; RT: rising time.

|  | individuals | notes | DF (Hz) | ND (s) | RT (s) |
| --- | --- | --- | --- | --- | --- |
|  |  |  |  |  |  |
| *obstetricans* allopatric | 27 | 156 | 1319.6 ± 133.1 (1119.7–1636.5) | 0.094 ± 0.020 (0.065–0.152) | 0.008 ± 0.002 (0.003–0.016) |
| *almogavarii* allopatric | 22 | 120 | 1431.9 ± 114.4 (1206.0–1636.5) | 0.090 ± 0.014 (0.071–0.144) | 0.009 ± 0.003 (0.001–0.019) |
| *obstetricans* parapatric | 9 | 52 | 1261.4 ± 41.2 (1205.8–1335.1) | 0.084 ± 0.012 (0.069–0.111) | 0.011 ± 0.004 (0.004–0.019) |
| *almogavarii* parapatric | 13 | 78 | 1354.9 ± 79.3 (1248.9–1507.3) | 0.086 ± 0.009 (0.064–0.109) | 0.008 ± 0.003 (0.005–0.022) |
|  |  |  |  |  |  |
|  |  |  |  |  |  |
